# Genetic risk impacts the association of menopausal hormone therapy with colorectal cancer risk

**DOI:** 10.1038/s41416-024-02638-2

**Published:** 2024-04-01

**Authors:** Yu Tian, Yi Lin, Conghui Qu, Volker Arndt, James W. Baurley, Sonja I. Berndt, Stephanie A. Bien, D. Timothy Bishop, Hermann Brenner, Daniel D. Buchanan, Arif Budiarto, Peter T. Campbell, Robert Carreras-Torres, Graham Casey, Andrew T. Chan, Rui Chen, Xuechen Chen, David V. Conti, Virginia Díez-Obrero, Niki Dimou, David A. Drew, Jane C. Figueiredo, Steven Gallinger, Graham G. Giles, Stephen B. Gruber, Marc J. Gunter, Sophia Harlid, Tabitha A. Harrison, Akihisa Hidaka, Michael Hoffmeister, Jeroen R. Huyghe, Mark A. Jenkins, Kristina M. Jordahl, Amit D. Joshi, Temitope O. Keku, Eric Kawaguchi, Andre E. Kim, Anshul Kundaje, Susanna C. Larsson, Loic Le Marchand, Juan Pablo Lewinger, Li Li, Victor Moreno, John Morrison, Neil Murphy, Hongmei Nan, Rami Nassir, Polly A. Newcomb, Mireia Obón-Santacana, Shuji Ogino, Jennifer Ose, Bens Pardamean, Andrew J. Pellatt, Anita R. Peoples, Elizabeth A. Platz, John D. Potter, Ross L. Prentice, Gad Rennert, Edward A. Ruiz-Narvaez, Lori C. Sakoda, Robert E. Schoen, Anna Shcherbina, Mariana C. Stern, Yu-Ru Su, Stephen N. Thibodeau, Duncan C. Thomas, Konstantinos K. Tsilidis, Franzel J. B. van Duijnhoven, Bethany Van Guelpen, Kala Visvanathan, Emily White, Alicja Wolk, Michael O. Woods, Anna H. Wu, Ulrike Peters, W. James Gauderman, Li Hsu, Jenny Chang-Claude

**Affiliations:** 1 School of Public Health, Capital Medical University, Beijing, China; 2 Division of Cancer Epidemiology, German Cancer Research Center (DKFZ), Heidelberg, Germany; 3 Public Health Sciences Division, Fred Hutchinson Cancer Research Center, Seattle, WA USA; 4 Division of Clinical Epidemiology and Aging Research, German Cancer Research Center (DKFZ), Heidelberg, Germany; 5 Bioinformatics and Data Science Research Center, Bina Nusantara University, Jakarta, Indonesia; 6 BioRealm LLC, Walnut, CA USA; 7 Division of Cancer Epidemiology and Genetics, National Cancer Institute, National Institutes of Health, Bethesda, MD USA; 8 Leeds Institute of Cancer and Pathology, University of Leeds, Leeds, UK; 9 Division of Preventive Oncology, German Cancer Research Center (DKFZ) and National Center for Tumor Diseases (NCT), Heidelberg, Germany; 10 German Cancer Consortium (DKTK), German Cancer Research Center (DKFZ), Heidelberg, Germany; 11 Colorectal Oncogenomics Group, Department of Clinical Pathology, The University of Melbourne, Parkville, VIC 3010 Australia; 12 University of Melbourne Centre for Cancer Research, Victorian Comprehensive Cancer Centre, Parkville, VIC 3010 Australia; 13 Genomic Medicine and Family Cancer Clinic, The Royal Melbourne Hospital, Parkville, VIC Australia; 14 Department of Epidemiology and Population Health, Albert Einstein College of Medicine, Bronx, NY USA; 15 Colorectal Cancer Group, ONCOBELL Program, Bellvitge Biomedical Research Institute (IDIBELL), L’Hospitalet de Llobregat, Barcelona, Spain; 16 Oncology Data Analytics Program, Catalan Institute of Oncology, L’Hospitalet de Llobregat, Barcelona, Spain; 17 Consortium for Biomedical Research in Epidemiology and Public Health (CIBERESP), Madrid, Spain; 18 Digestive Diseases and Microbiota Group, Girona Biomedical Research Institute Dr Josep Trueta (IDIBGI), Salt, 17190 Girona Spain; 19 Center for Public Health Genomics, University of Virginia, Charlottesville, VA USA; 20 Clinical and Translational Epidemiology Unit, Massachusetts General Hospital and Harvard Medical School, Boston, MA USA; 21 Division of Gastroenterology, Massachusetts General Hospital and Harvard Medical School, Boston, MA USA; 22 Channing Division of Network Medicine, Brigham and Women’s Hospital and Harvard Medical School, Boston, MA USA; 23 Broad Institute of Harvard and MIT, Cambridge, MA USA; 24 Department of Epidemiology, Harvard T.H. Chan School of Public Health, Harvard University, Boston, MA USA; 25 Department of Immunology and Infectious Diseases, Harvard T.H. Chan School of Public Health, Harvard University, Boston, MA USA; 26 Division of Biostatistics, Department of Population and Public Health Sciences, Keck School of Medicine, University of Southern California, Los Angeles, CA USA; 27 Nutrition and Metabolism Branch, International Agency for Research on Cancer, Lyon, France; 28 Department of Medicine, Samuel Oschin Comprehensive Cancer Institute, Cedars-Sinai Medical Center, Los Angeles, CA USA; 29 Department of Population and Public Health Sciences, Keck School of Medicine, University of Southern California, Los Angeles, CA USA; 30 Lunenfeld Tanenbaum Research Institute, Mount Sinai Hospital, University of Toronto, Toronto, ON Canada; 31 Centre for Epidemiology and Biostatistics, Melbourne School of Population and Global Health, The University of Melbourne, Melbourne, VIC Australia; 32 Cancer Epidemiology Division, Cancer Council Victoria, Melbourne, VIC Australia; 33 Precision Medicine, School of Clinical Sciences at Monash Health, Monash University, Clayton, VIC Australia; 34 Department of Medical Oncology & Therapeutics Research, City of Hope National Medical Center, Duarte, CA USA; 35 Department of Epidemiology and Biostatistics, School of Public Health, Imperial College London, London, UK; 36 Department of Radiation Sciences, Oncology Unit, Umeå University, Umeå, Sweden; 37 Department of Epidemiology, School of Public Health, University of Washington, Seattle, WA USA; 38 Center for Gastrointestinal Biology and Disease, University of North Carolina, Chapel Hill, NC USA; 39 Department of Genetics, Stanford University, Stanford, CA USA; 40 Department of Computer Science, Stanford University, Stanford, CA USA; 41 Institute of Environmental Medicine, Karolinska Institute, Stockholm, Sweden; 42 University of Hawaii Cancer Center, Honolulu, HI USA; 43 Department of Family Medicine, University of Virginia, Charlottesville, VA USA; 44 Department of Clinical Sciences, Faculty of Medicine and health Sciences and Universitat de Barcelona Institute of Complex Systems (UBICS), University of Barcelona (UB), L’Hospitalet de Llobregat, Barcelona, Spain; 45 Department of Global Health, Richard M. Fairbanks School of Public Health, Indianapolis, IN USA; 46 Department of Epidemiology, Richard M. Fairbanks School of Public Health, Indianapolis, Indianapolis, IN USA; 47 Department of Pathology, School of Medicine, Umm Al-Qura’a University, Mecca, Saudi Arabia; 48 Program in MPE Molecular Pathological Epidemiology, Department of Pathology, Brigham and Women’s Hospital, Harvard Medical School, Boston, MA USA; 49 Tokyo Medical and Dental University (Institute of Science Tokyo), Tokyo, Japan; 50 Huntsman Cancer Institute, Salt Lake City, UT USA; 51 Department of Population Health Sciences, University of Utah, Salt Lake City, UT USA; 52 Hochschule Hannover, University of Applied Sciences and Arts, Department III: Media, Information and Design, Hannover, Germany; 53 Department of Cancer Medicine, University of Texas MD Anderson Cancer Center, Houston, TX USA; 54 Department of Epidemiology, Johns Hopkins Bloomberg School of Public Health, Baltimore, MD USA; 55 Research Centre for Hauora and Health, Massey University, Wellington, New Zealand; 56 Department of Biostatistics, University of Washington, Seattle, WA USA; 57 Department of Community Medicine and Epidemiology, Lady Davis Carmel Medical Center, Haifa, Israel; 58 Ruth and Bruce Rappaport Faculty of Medicine, Technion-Israel Institute of Technology, Haifa, Israel; 59 Clalit National Cancer Control Center, Haifa, Israel; 60 Department of Nutritional Sciences, University of Michigan School of Public Health, Ann Arbor, MI USA; 61 Division of Research, Kaiser Permanente Northern California, Oakland, CA USA; 62 Department of Medicine and Epidemiology, University of Pittsburgh Medical Center, Pittsburgh, PA USA; 63 Biomedical Informatics Program, Department of Biomedical Data Sciences, Stanford University, Stanford, CA USA; 64 Kaiser Permanente Washington Health Research Institute, Seattle, WA USA; 65 Division of Laboratory Genetics, Department of Laboratory Medicine and Pathology, Mayo Clinic, Rochester, MN USA; 66 Department of Hygiene and Epidemiology, University of Ioannina School of Medicine, Ioannina, Greece; 67 Division of Human Nutrition and Health, Wageningen University & Research, Wageningen, The Netherlands; 68 Wallenberg Centre for Molecular Medicine, Umeå University, Umeå, Sweden; 69 Memorial University of Newfoundland, Discipline of Genetics, St. John’s, NL Canada; 70 University Cancer Centre Hamburg (UCCH), University Medical Centre Hamburg-Eppendorf, Hamburg, Germany

**Keywords:** Cancer epidemiology, Colorectal cancer, Cancer genetics

## Abstract

**Background:**

Menopausal hormone therapy (MHT), a common treatment to relieve symptoms of menopause, is associated with a lower risk of colorectal cancer (CRC). To inform CRC risk prediction and MHT risk-benefit assessment, we aimed to evaluate the joint association of a polygenic risk score (PRS) for CRC and MHT on CRC risk.

**Methods:**

We used data from 28,486 postmenopausal women (11,519 cases and 16,967 controls) of European descent. A PRS based on 141 CRC-associated genetic variants was modeled as a categorical variable in quartiles. Multiplicative interaction between PRS and MHT use was evaluated using logistic regression. Additive interaction was measured using the relative excess risk due to interaction (RERI). 30-year cumulative risks of CRC for 50-year-old women according to MHT use and PRS were calculated.

**Results:**

The reduction in odds ratios by MHT use was larger in women within the highest quartile of PRS compared to that in women within the lowest quartile of PRS (*p*-value = 2.7 × 10^−8^). At the highest quartile of PRS, the 30-year CRC risk was statistically significantly lower for women taking any MHT than for women not taking any MHT, 3.7% (3.3%–4.0%) vs 6.1% (5.7%–6.5%) (difference 2.4%, *P*-value = 1.83 × 10^−14^); these differences were also statistically significant but smaller in magnitude in the lowest PRS quartile, 1.6% (1.4%–1.8%) vs 2.2% (1.9%–2.4%) (difference 0.6%, *P*-value = 1.01 × 10^−3^), indicating 4 times greater reduction in absolute risk associated with any MHT use in the highest compared to the lowest quartile of genetic CRC risk.

**Conclusions:**

MHT use has a greater impact on the reduction of CRC risk for women at higher genetic risk. These findings have implications for the development of risk prediction models for CRC and potentially for the consideration of genetic information in the risk-benefit assessment of MHT use.

## Introduction

Colorectal cancer (CRC) is a commonly diagnosed malignancy that ranks third and second in terms of incidence and mortality, respectively, in the world [1]. Genome-wide association studies (GWAS) have identified a large number of genetic risk variants for CRC [2–4]. Aggregating genetic risk variants into a polygenic risk score (PRS) yields a continuous and quantitative measure of the estimated genetic predisposition to a certain disease at the individual level, which could be used to evaluate the impact of particular treatments or lifestyle modifications in individuals with high genetic risk [5].

Menopausal hormone therapy (MHT) is a common and effective treatment for relieving common symptoms of menopause for postmenopausal women, with a rapidly growing multibillion USD global market size [6]. Since the introduction of MHT use in the 1960s, it has been met with very high popularity until the publication of the Women’s Health Initiative (WHI) in 2002, which warned of serious health risks of MHT particularly in relation to breast cancer and cardiovascular disease, resulting in a dramatic decline in MHT use [7, 8]. In the following years, the use of MHT has gradually increased and is expected to further increase as some clinicians have raised the awareness of benefits of MHT potentially outweighing risks for some women’s health based on the women’s individual risk profile [9]. Currently, weighing the benefits and risks for personalized MHT treatment decisions does not take into account of genetic risk; however, it is expected that this would change in the future.

Since the first associations between MHT and CRC were made in the 1980s [10, 11], MHT use has been consistently shown to be associated with a reduced risk of CRC. A meta-analysis including 20 studies reported that both ever-use of estrogen-only MHT (RR: 0.79, 95% CI: 0.69–0.91) and ever-use of combined estrogen-progestogen MHT (RR: 0.74, 95% CI: 0.68–0.81) were associated with a reduced CRC risk [12]. Randomized controlled trial data from the Women’s Health Initiative indicated a lower risk of CRC among women taking estrogen plus progestin and no difference in CRC risk among users of estrogen-only, compared to placebo [13, 14].

Most studies of biological mechanisms have suggested that the protective cellular effect of MHT on CRC is likely to be mediated through nuclear estrogen receptors (i.e., ERα, ERβ) and progesterone receptor, which may involve increasing DNA repair, selectively activating proapoptotic signaling, inhibiting expression of oncogenes, regulating cell cycle progression, changing the miRNA pool and DNA methylation [15]. Nevertheless, these underlying etiologic mechanisms are not fully understood. Further insight into potential biological pathways could be gained by investigating genetic modifiers of CRC risk associated with MHT use. Through a genome-wide association study of gene-environment interaction, we previously identified genetic variants (*GRIN2B*, *DCBLD1*) that modified CRC risk associated with MHT use, offering new insights into pathways of CRC carcinogenesis and potential mechanisms involved [16].

CRC is a complex disease resulting from both genetic predisposition and environmental factors [17]. However, it is not yet known whether a genetic risk profile modifies the effect of MHT on CRC risk, i.e., whether there is an interaction between PRS and MHT. For a disease trait, interaction can be commonly described in two ways: multiplicative and additive. Multiplicative interaction focuses on the comparison of relative risk of an exposure (e.g. MHT) for one subgroup compared to another (e.g. high vs. low PRS). Analysis of multiplicative interaction can be performed directly using logistic regression and is typically considered the relevant scale for informing biological etiology. Additive interaction implies the difference in absolute risk due to exposure between one subgroup and another, and can improve the ability to identify relevant subgroups who may benefit the most from public health intervention, which is often neglected in epidemiologic studies. Finding an additive interaction can help guide public health campaigns aimed at identifying sub-populations in whom a specific intervention can lead to the greatest reduction in numbers of new cases, for example, women with high genetic susceptibility may have a greater benefit of reducing CRC risk with MHT use. Given that different information can be gained from studying different types of interactions, it is recommended to present both additive and multiplicative interaction in practice [18]. We therefore aimed to evaluate the joint associations of MHT and a PRS of 141 single nucleotide polymorphisms (SNPs) identified by previous GWAS with CRC risk and to assess both multiplicative and additive measures of interaction [2–4, 19–37]. Additionally, absolute risks were estimated for informing CRC prevention.

## Methods

### Study participants

We included studies from North America, Australia, and Europe participating in the multi-centered Colon Cancer Family Registry (CCFR), the Colorectal Transdisciplinary Study (CORECT), and the Genetics and Epidemiology of Colorectal Cancer Consortium (GECCO), all with GWAS data available, as previously described [4, 38, 39]. Study details and descriptions can be found in the supplementary section.

Cases were identified as incident invasive colorectal cancer cases and confirmed by medical records, pathological reports, or death certificate information. For cohort studies, nested case-control sets were assembled via risk-set sampling, while population-based controls were used for case-control studies. Controls were matched with cases on age and enrollment date, where applicable.

All studies were approved by their respective Institutional Review Boards, and all study participants provided informed consent.

### Exposure assessment

Information on demographics and environmental risk factors was collected by interview and/or structured questionnaire. We carried out a multi-step data-harmonization procedure at the GECCO coordinating center (Fred Hutchinson Cancer Research Center) as described previously [40–42].

Postmenopausal status was defined by using: (I) menopausal status derived from studies, if available; or (II) self-reported menopausal status, if study-derived was not available; or (III) age >55, if neither study-derived nor self-report were available. MHT use was considered using three variables, i.e., any MHT use, estrogen-only use, and combined estrogen-progestogen use at or until the reference date (date of diagnosis for cases, date of interview for controls). Estrogen-only use and combined estrogen-progestogen use were defined to be mutually exclusive, such that for example, combined estrogen-progestogen use excludes the use of estrogen-only or any other MHT at or until the reference time. Non-users of any MHT at or until the reference time were used as the reference group for all three MHT variables. For nested case-control studies from cohorts, the information on MHT use was collected at the enrollment date which was used as reference date. For case-control studies, the information collected on MHT use and duration for cases typically referred to use until diagnosis year or one to two years before diagnosis, depending on the individual studies; controls in case-control studies were similarly requested to provide information about MHT use until the time of recruitment/interview or the past 1–2 years to be consistent with assessment in cases (Supplementary Table 1).

### Genotyping, quality control, and imputation

Details on genotyping, imputation, and quality control have been reported previously [2]. In brief, genotyped SNPs were excluded on the basis of call rate (<98%) or evidence of departure from Hardy-Weinberg equilibrium (HWE) in controls (*P* < 1 × 10^−4^). For all studies, all autosomal SNPs were imputed to the Haplotype Reference Consortium r1.1 (2016) reference panel via the Michigan Imputation Server [43] and converted into a binary format for data management and analyses using R package BinaryDosage [44]. Imputed common SNPs were restricted based on a pooled MAF ≥ 1% and imputation accuracy (R^2^ > 0.8). All analyses were restricted to samples clustering with the Utah residents of Northern and Western European ancestry from the CEU population in principal component analysis.

### Derivation of polygenic risk score

The PRS was built based on 141 risk variants identified in previous GWAS of CRC risk (Supplementary Table 2) [2–4, 19–37]. The variant-specific weights were determined by the log-odds ratios estimated from prior studies. PRS was calculated by summing the product of the weight and the number of risk alleles for each risk variant across 141 identified genetic risk variants for all study participants. For the known variants identified by GECCO, CCFR, and CORECT studies, the estimates adjusted for winner’s curse [45] (i.e., a statistical effect resulting in the exaggeration of SNP-trait association estimates in the discovery study compared to their true association) were used. We employed quartiles of PRS (PRS.Q) as a categorical variable, using the lowest quartile as the reference group.

### Statistical analysis

Statistical analyses were conducted centrally on individual-level data. Logistic regression models were used to assess the association of PRS and MHT with CRC risk by odds ratios (ORs) and 95% confidence intervals (CIs) adjusted for age at the reference time, BMI, study, and the first three principal components to account for potential population substructure. *P*-values for trend in risks associated with quartiles of PRS were estimated by including the ordinal PRS.Q variable as a continuous variable in the regression models and testing coefficients using the Wald test. Heterogeneity *P*-values were calculated using Cochran’s Q statistics in study-specific meta-analyses [46].

We assessed multiplicative interaction effects of PRS and MHT variables by taking the products of PRS.Q and MHT variables in logistic regression models and obtained *P*-values using the likelihood ratio test. We assessed additive interaction effects by the relative excess risk due to interaction (RERI), i.e., departure of the joint effect of PRS and MHT variables from the sum of effect estimates for the two variables, and estimated the variance of RERI by the Delta method [47].

We also calculated the 30-year cumulative risk (%) of CRC for 50-year-old women according to MHT use and PRS to estimate the probability of developing CRC over a 30-year interval from age 50 to 80 years [48]. Specifically, we estimated the age-specific relative risks and attributable risks by three subgroups (≤60 years, 61–70 years, and >70 years) and combined these estimates with CRC incidence rates obtained from the SEER Research Data, 13 Registries, Nov 2019 Sub (1992–2017) [49] for White women only to obtain the baseline age-specific CRC hazard rates. We calculated the absolute risk for any given risk profile of MHT use and PRS, accounting for competing risks from non-CRC mortality rates which were obtained from the National Center for Health Statistics (https://seer.cancer.gov/mortality/). The 95% confidence intervals for absolute risk estimates were calculated based on 100 bootstrap samples.

All analyses were performed using SAS, version 9.4 (SAS Institute Inc, Cary, NC), and R, version 2.15.3 (R Foundation for Statistical Computing, Vienna, Austria) software. A two-sided *P*-value < 0.05 was considered statistically significant.

## Results

### Study population

The study sample for analysis comprised 28,486 post-menopausal women (11,519 cases and 16,967 controls) with genotype data and information on the use of any MHT, of which 10,027 women (35.2%) indicated the use of any MHT. A total of 7637 women provided information on the use of estrogen-only and 6887 women on combined estrogen-progestogen use. Among these women, 2156 (28.2%) used estrogen-only and 1509 (21.9%) used combined estrogen-progestogen. Detailed descriptive characteristics of the cases and controls are shown in Supplementary Table 3.

### Association of MHT or PRS with CRC risk

MHT use was associated with a reduced CRC risk in our pooled analyses. Compared to non-users, the OR for CRC was 0.71 (95% CI: 0.64–0.78, Supplementary Fig. 1) for women using any MHT, 0.65 (95% CI: 0.53–0.79, Supplementary Fig. 2) for women using estrogen-only, and 0.73 (95% CI: 0.59–0.90, Supplementary Fig. 3) for women using combined estrogen-progestogen. The risk reduction of CRC associated with MHT use is consistent in both cohort and case-control studies (Supplementary Figs. 1–3). The risk for CRC increased with higher quartiles of PRS compared to the lowest quartile [ORs, for PRS.Q2: 1.49 (1.43–1.55); PRS.Q3: 1.92 (1.84–2.00); PRS.Q4: 2.87 (2.76–2.99)].

### Joint associations of MHT and PRS with CRC risk

**Fig. 1:**
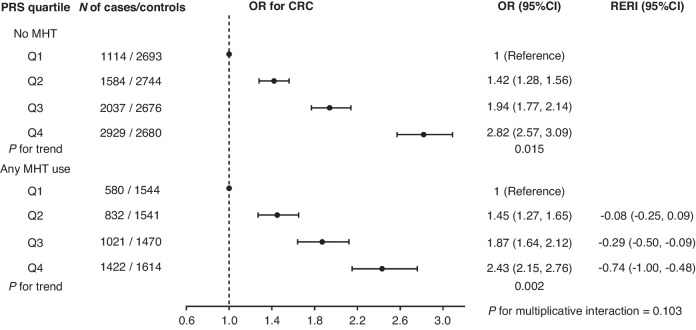
Effects of polygenic risk score and menopausal hormone therapy on colorectal cancer risk. *PRS.Q* the quartiles of polygenic risk score, *OR* odds ratio, 95%CI 95% confidence interval, *CRC* colorectal cancer, *MHT* menopausal hormone therapy, *RERI* the relative excess risk due to interaction. The regression model was adjusted for age, BMI, study center, and the first three principal components.

There was a pattern of higher CRC risk with higher quartiles of PRS for both users and non-users of MHT, with a significant linear trend across quartiles of PRS (No MHT use, *P* for trend = 0.015; MHT use, *P* for trend = 0.002) (Fig. 1). The increased risks of CRC associated with PRS seemed to be similar in non-users and users of MHT within the same PRS.Q, e.g., for the highest vs lowest PRS quartile, ORs were 2.82 (2.57, 3.09) in non-users and 2.43 (2.15, 2.76) in users of any MHT (Table 1). Similar patterns were also observed for the use of estrogen-only (Table 1, Supplementary Fig. 4) and combined estrogen-progestogen (Table 1, Supplementary Fig. 5).

**Table 1:** Associations of all MHT variables with CRC risk stratified by quartiles of PRS.

	Quartiles of PRS [OR (95%CI)]						
	PRS.Q1	PRS.Q2	PRS.Q3	PRS.Q4	Q2 within strata of MHT	Q3 within strata of MHT	Q4 within strata of MHT
No MHT	1 (ref.)	**1.42 (1.28, 1.56)**	**1.94 (1.77, 2.14)**	**2.82 (2.57, 3.09)**	**1.42 (1.28, 1.56)**	**1.94 (1.77, 2.14)**	**2.82 (2.57, 3.09)**
Any MHT	**0.75 (0.66, 0.85)**	1.09 (0.97, 1.22)	**1.40 (1.25, 1.57)**	**1.83 (1.64, 2.04)**	**1.45 (1.27, 1.65)**	**1.87 (1.64, 2.12)**	**2.43 (2.15, 2.76)**
Within strata of PRS.Q	**0.75 (0.66, 0.85)**	**0.77 (0.69, 0.86)**	**0.72 (0.65, 0.80)**	**0.65 (0.59, 0.72)**	*P* for multiplicative interaction = 0.103		
	RERI	−0.08 (−0.25, 0.09)	**−0.29 (−0.50, −0.09)**	**−0.74**^**a**^ **(−1.00, −0.48)**			
No MHT	1 (ref.)	**1.35 (1.14, 1.60)**	**1.84 (1.57, 2.17)**	**2.35 (2.00, 2.75)**	**1.35 (1.14, 1.60)**	**1.84 (1.57, 2.17)**	**2.35 (2.00, 2.75)**
E-only	**0.78 (0.62, 0.98)**	0.91 (0.73, 1.13)	1.20 (0.97, 1.47)	**1.37 (1.12, 1.68)**	1.16 (0.89, 1.52)	**1.53 (1.19, 1.99)**	**1.76 (1.37, 2.26)**
Within strata of PRS.Q	**0.78 (0.62, 0.98)**	**0.67 (0.54, 0.83)**	**0.65 (0.53, 0.79)**	**0.58 (0.48, 0.71)**	*P* for multiplicative interaction = 0.302		
	RERI	−0.22 (−0.53, 0.09)	**−0.43 (−0.79, −0.06)**	**−0.76 (−1.17, −0.34)**			
No MHT	1 (ref.)	**1.37 (1.16, 1.62)**	**1.86 (1.58, 2.19)**	**2.35 (2.00, 2.76)**	**1.37 (1.16, 1.62)**	**1.86 (1.58, 2.19)**	**2.35 (2.00, 2.76)**
E + P	0.77 (0.59, 1.01)	1.02 (0.79, 1.31)	**1.37 (1.08, 1.73)**	**1.59 (1.27, 2.00)**	1.32 (0.96, 1.83)	**1.77 (1.30, 2.42)**	**2.07 (1.53, 2.80)**
Within strata of PRS.Q	0.77 (0.59, 1.01)	**0.74 (0.58, 0.95)**	**0.73 (0.59, 0.92)**	**0.68 (0.55, 0.84)**	*P* for multiplicative interaction = 0.886		
	RERI	−0.12 (−0.48, 0.23)	−0.27 (−0.68, 0.15)	**−0.53 (−1.00, −0.07)**			

The regression models were adjusted for age, BMI, study center, and the first three principal components. Statistically significant values are indicated in bold.

The cells of columns “PRS.Q” intersecting rows “Any MHT” or “No MHT” present the ORs of CRC for MHT users or non-users with different PRS.Q using non-users with PRS.Q1 as reference. ORs of CRC for women with different PRS.Q to those with PRS.Q1 stratified by the use of MHT are presented in the right three columns “Q within strata of MHT”. ORs of CRC for MHT users to non-users stratified by PRS.Q are presented in the row “Within strata of PRS.Q”.

*CRC* colorectal cancer, *PRS.Q* the quartiles of polygenic risk score, *OR* odds ratio, *95%CI* 95% confidence interval, *MHT* menopausal hormone therapy, *E-only* estrogen-only therapy, *E* *+* *P* combined estrogen-progestogen therapy, *RERI* the relative excess risk due to interaction.

^a^RERI = −0.74, which equals to OR_11_-OR_10_-OR_01_ + 1 = 1.83–2.82–0.75 + 1, means that the protective effect of MHT use is stronger in the highest quartile of PRS with 74% relative risk reduction more compared to that in the lowest quartile of PRS.

The reduction in odds ratio by MHT use was however stronger in women within the highest quartile of PRS [OR = 0.65 (0.59–0.72)] than that in women within the lowest quartile of PRS [0.75 (0.66–0.85)]. Similar patterns were found for joint associations of MHT types (estrogen-only, combined estrogen-progesterone) and PRS. For all three MHT variables, there was no significant multiplicative interaction with PRS (all *P*-values > 0.05). However, we observed statistically significant additive interactions consistently across all three MHT variables for the highest quartile of genetic risk [RERI: −0.74 (−1.00, −0.48), *P*-value = 2.7 ×10^−8^ for any MHT use; RERI: −0.76 (−1.17, −0.34), *P*-value = 3.8 ×10^−4^ for estrogen-only use, and RERI: −0.53 (−1.00, −0.07), *P*-value = 0.025 for combined estrogen-progestogen use] when compared to the risk excess reductions due to MHT use in those at the lowest quartile of PRS (Table 1, Fig. 1, Supplementary Figs. 4 and 5). In other words, the joint effect of MHT use and high genetic susceptibility on CRC risk differed significantly from that expected from the sum of the individual effects.

We have further analyzed the joint association of MHT and PRS with colorectal cancer risk stratified by tumor anatomical sites (colon, rectum, proximal colon, and distal colon), and observed to some extent statistically significant additive interaction between PRS and all three MHT variables for the different tumor sites (Supplementary Tables 4–7). The magnitudes of RERI for quartiles of PRS across all three MHT variables were more pronounced for risk of distal colon (e.g., −0.35 to −1.12 for any MHT use) than proximal colon (e.g., −0.05 to −0.51 for any MHT use).

### Absolute risk estimates for CRC by MHT and PRS

**Fig. 2:**
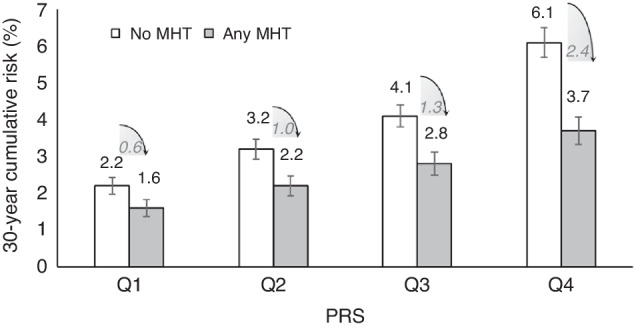
The 30-year cumulative risk estimates (%) of CRC for 50-year-old women, according to any MHT use and quartiles of PRS. *CRC* colorectal cancer, *PRS.Q* the quartiles of polygenic risk score, *MHT* menopausal hormone therapy.

The projected 30-year cumulative risks of CRC for 50-year-old women who used any MHT were consistently lower than those for non-users across quartiles of PRS. The difference in 30-year cumulative risk between users of any MHT and non-users increased with higher quartiles of PRS, implying a greater risk reduction effect of MHT for women at higher genetic risk. At the highest quartile of PRS, the 30-year CRC risk was statistically significantly lower for women taking any MHT than for women not taking any MHT, 3.7% (3.3%–4.0%) vs 6.1% (5.7%–6.5%) (difference 2.4%, *P*-value = 1.83 ×10^−14^); these differences were also statistically significant but smaller in magnitude in the lowest PRS quartile, 1.6% (1.4%–1.8%) vs 2.2% (1.9%–2.4%) (difference 0.6%, *P*-value = 1.01 ×10^−3^) (Table 2, Fig. 2). The reduction in absolute risk associated with any MHT use was thus 4 times greater in the highest versus lowest quartile of genetic risk (2.4% vs 0.6%, Fig. 2). Similar patterns for 30-year cumulative risks of CRC for 50-year-old women according to quartiles of PRS were also found for estrogen-only use and combined estrogen-progestogen use, respectively (Table 2, Supplementary Figs. 6 and 7).

**Table 2:** 30-year cumulative risk estimates (%) of CRC for 50-year-old women by use of all MHT variables and quartiles of PRS.

	30-year absolute risk, % (95% CI)							
	Ca/Co, *n*	PRS.Q1	Ca/Co, *n*	PRS.Q2	Ca/Co, *n*	PRS.Q3	Ca/Co, *n*	PRS.Q4
No MHT	1114/2693	2.16 (1.94, 2.39)	1584/2744	3.15 (2.86, 3.43)	2037/2676	4.13 (3.82, 4.43)	2929/2680	6.06 (5.66, 6.46)
Any MHT	580/1544	1.59 (1.36, 1.82)	832/1541	2.20 (1.93, 2.47)	1021/1470	2.83 (2.51, 3.14)	1422/1614	3.66 (3.29, 4.03)
*Diff*		*0.57*		*0.95*		*1.30*		*2.40*
*P* value		1.01 × 10^−3^		3.65 × 10^−5^		3.27 × 10^−8^		1.83 × 10^−14^
No MHT	447/628	2.45 (2.10, 2.80)	630/661	3.04 (2.70, 3.38)	776/593	4.23 (3.81, 4.65)	1088/658	5.32 (4.83, 5.81)
E-Only	157/279	1.88 (1.44, 2.32)	199/317	1.99 (1.55, 2.44)	253/285	2.95 (2.28, 3.62)	322/344	3.13 (2.45, 3.80)
*Diff*		*0.57*		*1.05*		*1.28*		*2.19*
*P* value		7.93 × 10^−2^		2.85 × 10^−4^		6.39 × 10^−3^		7.26 × 10^−6^
No MHT	438/618	2.34 (2.03, 2.64)	622/649	2.82 (2.45, 3.18)	760/583	4.06 (3.60, 4.53)	1064/644	4.97 (4.54, 5.40)
E + P	100/188	1.89 (0.97, 2.81)	155/207	2.22 (1.26, 3.17)	187/193	2.76 (2.00, 3.52)	256/223	4.11 (2.73, 5.49)
*Diff*		*0.45*		*0.60*		*1.30*		*0.86*
*P* value		0.44		0.35		4.56 × 10^−3^		0.32

*CRC* colorectal cancer, *Ca/Co* number of cases patients and controls individuals, *PRS.Q* the quartiles of polygenic risk score, *95% CI* 95% confidence interval, *MHT* menopausal hormone therapy, *E-only* estrogen-only therapy, *E* *+* *P* combined estrogen-progestogen therapy, *Diff* the estimated difference of absolute risks between MHT users and non-users, *P* an alpha level of 0.05 (two-sided) was considered to be statistically significant for comparing the absolute risks of MHT users to those of non-users.

After stratifying by tumor anatomical sites, we further observed that the reduction in absolute risk associated with MHT use for women with higher genetic risk compared to the lowest quartile of PRS was somewhat greater for distal colon cancer (e.g., 0.22%, 0.30%, and 0.76% for any MHT use) than that for proximal colon cancer (e.g., 0.17%, 0.31%, and 0.62% for any MHT use) (Supplementary Tables 8–11).

## Discussion

Based on a large sample size derived from international colorectal cancer consortia, we observed statistically significant modification of MHT associated CRC risk by genetic risk for this disease, which was evidenced by substantial additive interactions between PRS and MHT variables on CRC risk. As such, the reduction in 30-year absolute risk of developing CRC as a result of MHT use was more apparent among 50-year-old women with higher genetic risk profiles, showing that the genetically predetermined increased risk of CRC could be offset to some extent by the use of MHT.

Several previous studies have reported potential association of some genetic modifiers and MHT with CRC risk [40, 50–52]. However, to our knowledge, studies have not investigated associations of aggregated genetic susceptibility with MHT for CRC risk. Although some previous studies reported the joint association of PRS and environmental factors, including diet, lifestyle, and behavior factors, with the risk of CRC [53–61], these studies did not address potential interactions between PRS and MHT. Considering the high use, known risks of use, and the big market value of MHT globally, our study provides new insight on the association between MHT and CRC risk in people with different genetic susceptibilities.

Herein, our study found that MHT has a strong impact on reducing the risk of CRC which may differ by genetic factors, i.e., with increasing genetic susceptibility, women using MHT had a greater reduction in CRC risk compared to non-users. Nevertheless, based on these findings alone we do not simply advocate the use of MHT as a chemoprevention intervention in those with high genetic risk for CRC because of its potential adverse consequences with long-term use of the increased risk of stroke [7] or breast cancer [8]. Instead, our study points to a potential future consideration of genetic risk in evaluating the risk-benefit assessment of MHT use. We do acknowledge that MHT remains widely used and as such, under the model of personalized medicine, it may be possible to use the genetic risk for CRC as input into decisions for or against MHT use when an individual woman is considering using MHT for other reasons such as menopausal symptoms or osteoporosis treatment.

We additionally found some differences in the joint associations of MHT and PRS with CRC risk according to anatomical site of the tumor. When additionally considering women’s PRS, MHT use appeared to have a slightly stronger protective effect across PRS on cancer occurring in the distal colon compared to the proximal colon, and correspondingly slightly stronger protective benefits with increasing PRS in terms of absolute risks. Prior studies indicated that MHT was associated with a stronger reduced cancer risk for the distal colon rather than the proximal colon, without consideration of PRS [62, 63]. The underlying mechanism remains uncertain but may be related to tumor heterogeneity in carcinogenic processes in different sites of the large bowel with different embryonic origins, somatic mutation profile, and microbiomes [64–68]. Further studies are needed to validate the observed tumor site differences and to determine the reasons why the association between MHT and CRC risk is attenuated for the proximal colon.

We investigated the association of PRS and MHT with CRC risk in postmenopausal women by using the largest number of 141 GWAS-identified genetic variants of CRC risk, resulting in a more comprehensive genetic score than any previous study, and with the largest sample size to date. We performed the assessment of both multiplicative and additive interaction, which may provide insight into the mechanisms of disease [18]. It is worth noting that for the gene-environment interaction studies focusing on single SNPs, there is little or no difference between additive and multiplicative interaction due to weak SNP effect size, as commonly observed [69]. However, when PRS is used to capture overall genetic susceptibility, the difference between multiplicative interaction and additive interaction (RERI) may be substantial. In our study, we found statistically significant additive interactions but not significant multiplicative interactions for MHT use. This observation indicates the importance of assessing interaction on both additive and multiplicative scales, where an additive interaction from a public health perspective is a desirable scale for risk stratification because it identifies sub-populations in whom a specific intervention can prevent the largest number of cancer occurrences; taking both genetic and MHT factors into account could be meaningful for making improved predictions for CRC risk as suggested by the results of our study [70].

To our knowledge, this is the first study to report on the joint association and interaction of CRC-related PRS and MHT variables with CRC risk, as well as with tumor site-specific risk, using both multiplicative and additive interaction. Our study also has several potential limitations. First, MHT information in some studies was self-reported; therefore, it may lead to recall bias (in the retrospective studies) or misclassification (in the prospective studies). However, previous studies have found a high validity for self-reported MHT use when compared with population-based prescription databases [71] and a high concordance between self-reported MHT use and physicians’ reports [72]. Second, because some studies asked only about current MHT use at the reference time rather than ever-use of MHT until the reference time, the status of MHT use might be misclassified, which would be likely to result in an underestimation of the strength of association. Third, the SNPs used for PRS as well as the study samples are population specific for postmenopausal women of European ancestries, thus generalization of results to populations of other racial and ethnic groups needs to be further evaluated.

In conclusion, the joint associations of genetic risk as measured by the PRS and the use of MHT with CRC risk show departures from the additive model. MHT use has a stronger impact on the risk reduction of CRC for women at higher genetic risk. These findings will inform the development of risk-prediction models for CRC in the future. They may lead to the consideration of genetic information as an additional factor in the risk-benefit assessment regarding MHT use in both the public health and clinical practice settings.

### Supplementary information

Supplemental material

Study description

## Data Availability

The genotype data as well as limited phenotype data have been deposited at dbGaP under accession numbers phs001415.v1.p1, phs001315.v1.p1, phs001078.v1.p1, phs001499.v1.p1, phs001903.v1.p1, phs001856.v1.p1, phs001045.v1.p1, and phs001499.v1.p1. Further information is available from the corresponding authors upon request.

## Code availability

The code, as a part of an international consortium, which was used to generate results of this article, can be accessed on reasonable request to the corresponding authors.
