# Supplementary material for: Genetic risk impacts the association of menopausal hormone therapy with colorectal cancer risk
Source: Br J Cancer. 2024 Apr 1;130(10):1687–96. doi: 10.1038/s41416-024-02638-2 (PMC11091089; doi:10.1038/s41416-024-02638-2)
Supplement: Supplementary file 1 — Supplemental material [file 41416_2024_2638_MOESM1_ESM.docx]

**Supplementary table 1.** The definitions of the menopausal status and the reference time for each included study

| **Study** | **Menopausal status** | **Reference time** |
| --- | --- | --- |
| **Cohort-based** |  |  |
| Campaign against Cancer and Heart Disease II (CLUEII) | Study defined | The year 1989 at recruitment to CLUE II cohort study |
| Cancer Prevention Study-II (CPSII) | Study defined | The year at which non-diet exposure information is either from the 1992 survey if a case was diagnosed before the 1997 survey (and its matched control), or from the 1997 survey if a case was diagnosed after the 1997 survey (and its matched control) |
| European Prospective Investigation into Cancer (EPIC) | Study defined | The year at recruitment |
| Melbourne Collaborative Cohort Study (MCCS) | Self-reported | The year at baseline attendance. |
| Multiethnic Cohort Study (MEC) | Study defined | The year at questionnaire |
| Nurses’ Health Study (NHS) | Self-reported | The year closest to blood draw |
| Prostate, Lung, Colorectal, and Ovarian Cancer Screening Trial (PLCO) | Study defined | The year at complete baseline questionnaire |
| Swedish Mammography Cohort and Swedish Men Cohort (SMC_COSM) | Self-reported | The year at diagnosis for cases or the year at selection for control |
| UK Biobank (UKB) | Self-reported | The year when attended assessment center |
| Cancer Screening Trial VITamins And Lifestyle cohort (VITAL) | Self-reported + study defined | The time of enrollment into the cohort |
| The Women's Health Initiative (WHI) | Criteria for enrollment, study defined | The time of enrollment into the cohort |
| **Case-control** |  |  |
| Colon Cancer Family Registry (CCFR) | Self-reported | Two years prior to diagnosis |
| Hawaii Colorectal Cancer Studies 2 & 3 (Colo 2&3) | Self-reported | The year at survey |
| Colorectal Cancer Genetics & Genomics (CRCGEN) | Self-reported | The year at diagnosis for cases or the year at selection for control |
| Darmkrebs: Chancen der Verhütung durch Screening (DACHS) | Study Derived | The year of diagnosis (cases) or the year of interview (controls) |
| Diet, Activity, and Lifestyle Study (DALS) | Study defined | The year at survey Study participants were asked to recall the year 2 years prior to the date of selection (i.e., the date of diagnosis for cases or the date of selection for controls) |
| Epidemiologische Studie zu Chancen der Verhütung, Früherkennung und optimierten THerapie chronischer ERkrankungen in der älteren Bevölkerung (ESTHER_VERDI) | Self-reported | The year at recruitment |
| Kentucky Case-Control Study (Kentucky) | Study defined | The year when entered study |
| Leeds Colorectal Cancer Study (LCCS) | No data from study, set >=55 year as postmenopausal | One year prior to diagnosis |
| Molecular Epidemiology of Colorectal Cancer Study (MECC) | Self-reported + study defined | The year at interview |
| The North Carolina Colon Cancer Study II (NCCCSII) | Self-reported | The year at interview |
| Newfoundland Case-Control Study (NFCCR) | Study defined | The time when questionnaire was completed |
| Colon Cancer Pathways: Hyperplastic Polyps and Adenomas (REACH) | Self-reported | The year at detection/indication |
| University of Southern California Hormone Replacement Therapy Colorectal Cancer Study (USC_HRT_CRC) | Self-reported | The year at diagnosis for cases or the year at selection for control |

**Supplementary table 2.** List of SNPs included in the polygenic risk score

| **SNP** | **Locus** | **Chr** | **Position  (hg19)** | **Risk  allele** | **RAF** | **Weight  (beta)** | ***P* value** | **PMID first reported** | **Author first reported** | **Year first  reported** |
| --- | --- | --- | --- | --- | --- | --- | --- | --- | --- | --- |
| rs72647484 | 1p36.12 | 1 | 22587728 | T | 0.91 | 0.050 | 1.50E-03 | 25990418 | Al-Tassan | 2015 |
| rs4360494 | 1p34.3 | 1 | 38455891 | G | 0.45 | 0.052 | 3.80E-09 | 30510241 | Huyghe | 2019 |
| rs12144319 | 1p32.3 | 1 | 55246035 | C | 0.25 | 0.067 | 3.30E-11 | 30510241 | Huyghe | 2019 |
| rs7542665 | 1p31.3 | 1 | 62673037 | C | 0.27 | 0.077 | 3.51E-08 | 30529582 | Lu | 2019 |
| rs6678517 | 1q25.3 | 1 | 183002639 | A | 0.59 | 0.073 | 2.40E-16 | 24737748 | Whiffin | 2014 |
| rs17011141 | 1q41 | 1 | 222112634 | G | 0.21 | 0.088 | 6.10E-16 | 20972440 | Houlston | 2010 |
| rs7606562 | 2p16.3 | 2 | 48686695 | T | 0.81 | 0.095 | 1.21E-08 | 30529582 | Lu | 2019 |
| rs11692435 | 2q11.2 | 2 | 98275354 | G | 0.90 | 0.113 | 1.22E-08 | 31089142 | Law | 2019 |
| rs448513 | 2q24.2 | 2 | 159964552 | C | 0.33 | 0.051 | 4.40E-08 | 30510241 | Huyghe | 2019 |
| rs11884596 | 2q33.1 | 2 | 199612407 | C | 0.38 | 0.054 | 5.00E-09 | 30510241 | Huyghe | 2019 |
| rs983402 | 2q33.1 | 2 | 199781586 | T | 0.33 | 0.063 | 3.70E-11 | 30510241 | Huyghe | 2019 |
| rs3731861 | 2q35 | 2 | 219191256 | T | 0.63 | 0.061 | 1.50E-11 | 27005424 | Orlando | 2016 |
| rs35470271 | 3p22.1 | 3 | 40915239 | G | 0.15 | 0.099 | 1.20E-16 | 26151821 | Schumacher | 2014 |
| rs9831861 | 3p21.1 | 3 | 53088285 | G | 0.59 | 0.068 | 4.17E-10 | 31089142 | Law | 2019 |
| rs6781752 | 3p14.1 | 3 | 66365163 | A | 0.21 | 0.060 | 7.10E-08 | 26151821 | Schumacher | 2015 |
| rs13086367 | 3q13.2 | 3 | 112903888 | A | 0.53 | 0.047 | 7.00E-08 | 30510241 | Huyghe | 2019 |
| rs12635946 | 3q13.2 | 3 | 112916918 | C | 0.62 | 0.077 | 1.02E-11 | 31089142 | Law | 2019 |
| rs72942485 | 3q13.2 | 3 | 112999560 | G | 0.98 | 0.176 | 1.40E-08 | 30510241 | Huyghe | 2019 |
| rs10049390 | 3q22.2 | 3 | 133701119 | A | 0.74 | 0.060 | 3.80E-09 | 30510241 | Huyghe | 2019 |
| rs113569514 | 3q22.2 | 3 | 133748789 | T | 0.62 | 0.095 | 2.45E-12 | 30529582 | Lu | 2019 |
| rs9876206 | 3q26.2 | 3 | 169517436 | C | 0.75 | 0.045 | 7.80E-06 | 20972440 | Houlston | 2010 |
| rs13149359 | 4q22.2 | 4 | 94938618 | A | 0.37 | 0.052 | 1.20E-08 | 29917119 | Schmit | 2019 |
| rs1391441 | 4q24 | 4 | 106128760 | A | 0.67 | 0.052 | 1.60E-08 | 30510241 | Huyghe | 2019 |
| rs11727676 | 4q31.21 | 4 | 145659064 | C | 0.10 | 0.084 | 2.90E-08 | 30510241 | Huyghe | 2019 |
| rs78368589 | 5p15.33 | 5 | 1240204 | T | 0.06 | 0.112 | 5.10E-09 | 30510241 | Huyghe | 2019 |
| rs2735940 | 5p15.33 | 5 | 1296486 | G | 0.50 | 0.087 | 1.40E-22 | 29917119 | Schmit | 2019 |
| rs7708610 | 5p13.1 | 5 | 40102443 | A | 0.36 | 0.055 | 4.20E-09 | 30510241 | Huyghe | 2019 |
| rs12514517 | 5p13.1 | 5 | 40280076 | A | 0.29 | 0.101 | 9.30E-25 | 29917119 | Schmit | 2019 |
| rs145364999 | 5q21.1 | 5 | 98206082 | T | 1.00 | 0.556 | 6.30E-09 | 30510241 | Huyghe | 2019 |
| rs755229494 | 5q22.2 | 5 | 112097351 | G | 0.00 | 0.629 | 2.10E-12 | 14624392 | Niell | 2003 |
| rs12659017 | 5q23.2 | 5 | 125988175 | G | 0.23 | 0.086 | 4.45E-08 | 30529582 | Lu | 2019 |
| rs4976270 | 5q31.1 | 5 | 134467220 | C | 0.55 | 0.069 | 4.80E-15 | 23263487 | Jia | 2013 |
| rs2070699 | 6p24.1 | 6 | 12292772 | T | 0.48 | 0.068 | 3.88E-09 | 31089142 | Law | 2019 |
| rs1476570 | 6p22.1 | 6 | 29809860 | A | 0.38 | 0.113 | 6.71E-09 | 30529582 | Lu | 2019 |
| rs3131043 | 6p21.33 | 6 | 30758466 | G | 0.43 | 0.068 | 2.67E-08 | 31089142 | Law | 2019 |
| rs116353863 | 6p21.33 | 6 | 31010185 | C | 0.02 | 0.155 | 8.30E-07 | 30510241 | Huyghe | 2019 |
| rs116685461 | 6p21.33 | 6 | 31315512 | G | 0.88 | 0.070 | 1.90E-07 | 30510241 | Huyghe | 2019 |
| rs2516420 | 6p21.33 | 6 | 31449620 | C | 0.93 | 0.112 | 1.80E-10 | 30510241 | Huyghe | 2019 |
| rs3830041 | 6p21.32 | 6 | 32191339 | T | 0.14 | 0.148 | 1.65E-08 | 30529582 | Lu | 2019 |
| rs9271695 | 6p21.32 | 6 | 32593080 | G | 0.80 | 0.089 | 4.90E-14 | 30510241 | Huyghe | 2019 |
| rs16878812 | 6p21.31 | 6 | 35569562 | A | 0.89 | 0.078 | 3.60E-08 | 29917119 | Schmit | 2019 |
| rs9470361 | 6p21.2 | 6 | 36623379 | A | 0.25 | 0.054 | 8.60E-08 | 22634755 | Dunlop | 2012 |
| rs62396735 | 6p21.1 | 6 | 41702582 | C | 0.29 | 0.033 | 6.30E-04 | 26965516 | Zeng | 2016 |
| rs13204733 | 6p12.1 | 6 | 55566108 | G | 0.14 | 0.068 | 1.50E-07 | 30510241 | Huyghe | 2019 |
| rs62404966 | 6p12.1 | 6 | 55712124 | C | 0.76 | 0.072 | 1.10E-11 | 29917119 | Schmit | 2019 |
| rs2079660 | 6q21 | 6 | 105970139 | C | 0.91 | 0.122 | 1.37E-08 | 31089142 | Law | 2019 |
| rs12672022 | 7p13 | 7 | 45136423 | T | 0.83 | 0.065 | 4.70E-08 | 30510241 | Huyghe | 2019 |
| rs80077929 | 7p12.3 | 7 | 46094089 | T | 0.11 | 0.064 | 8.50E-06 | 30510241 | Huyghe | 2019 |
| rs10951878 | 7p12.3 | 7 | 46926695 | C | 0.49 | 0.058 | 1.10E-08 | 31089142 | Law | 2019 |
| rs3801081 | 7p12.3 | 7 | 47511161 | G | 0.68 | 0.077 | 2.00E-11 | 31089142 | Law | 2019 |
| rs16892766 | 8q23.3 | 8 | 117630683 | C | 0.08 | 0.210 | 7.30E-28 | 26965516 | Zeng | 2016 |
| rs6469654 | 8q23.3 | 8 | 117632965 | G | 0.23 | 0.068 | 3.90E-08 | 18372905 | Tomlinson | 2008 |
| rs117079142 | 8q24.11 | 8 | 117790914 | A | 0.04 | 0.114 | 7.50E-07 | 26965516 | Zeng | 2016 |
| rs6983267 | 8q24.21 | 8 | 128413305 | G | 0.52 | 0.105 | 1.10E-15 | 17618284 | Tomlinson | 2007 |
| rs7013278 | 8q24.21 | 8 | 128414892 | T | 0.38 | 0.061 | 6.70E-06 | 30510241 | Huyghe | 2019 |
| rs4313119 | 8q24.21 | 8 | 128571855 | G | 0.75 | 0.061 | 1.80E-09 | 30510241 | Huyghe | 2019 |
| rs1537372 | 9p21.3 | 9 | 22103183 | G | 0.57 | 0.050 | 1.40E-08 | 30510241 | Huyghe | 2019 |
| rs34405347 | 9q22.33 | 9 | 101679752 | T | 0.90 | 0.082 | 3.10E-08 | 30510241 | Huyghe | 2019 |
| rs10980628 | 9q31.3 | 9 | 113671403 | C | 0.21 | 0.064 | 2.80E-09 | 30510241 | Huyghe | 2019 |
| rs12217641 | 10p14 | 10 | 8663875 | C | 0.70 | 0.046 | 6.80E-06 | 30510241 | Huyghe | 2019 |
| rs11255841 | 10p14 | 10 | 8739580 | T | 0.70 | 0.106 | 1.30E-25 | 18372905 | Tomlinson | 2008 |
| rs10821907 | 10q11.23 | 10 | 52648454 | C | 0.83 | 0.073 | 5.00E-10 | 29917119 | Schmit | 2019 |
| rs704017 | 10q22.3 | 10 | 80819132 | G | 0.58 | 0.077 | 1.80E-17 | 24836286 | Zhang | 2014 |
| rs1250567 | 10q22.3 | 10 | 81046265 | C | 0.44 | 0.048 | 6.80E-08 | 30510241 | Huyghe | 2019 |
| rs10786560 | 10q24.2 | 10 | 101315166 | G | 0.76 | 0.051 | 5.00E-06 | 30510241 | Huyghe | 2019 |
| rs11190164 | 10q24.2 | 10 | 101351704 | G | 0.26 | 0.089 | 1.00E-17 | 24737748 | Whiffin | 2014 |
| rs12246635 | 10q25.2 | 10 | 114288619 | C | 0.10 | 0.098 | 1.30E-11 | 25105248 | Wang | 2014 |
| rs11196170 | 10q25.2 | 10 | 114722621 | A | 0.22 | 0.053 | 7.00E-07 | 24836286 | Zhang | 2014 |
| rs4450168 | 11p15.4 | 11 | 10286755 | C | 0.17 | 0.095 | 1.24E-08 | 31089142 | Law | 2019 |
| rs174533 | 11q12.2 | 11 | 61549025 | G | 0.67 | 0.064 | 1.20E-11 | 24836286 | Zhang | 2014 |
| rs7121958 | 11q13.4 | 11 | 74280012 | G | 0.51 | 0.078 | 8.90E-19 | 22634755 | Dunlop | 2012 |
| rs7946853 | 11q13.4 | 11 | 74409077 | C | 0.86 | 0.060 | 3.30E-06 | 30510241 | Huyghe | 2019 |
| rs61389091 | 11q13.4 | 11 | 74427921 | C | 0.96 | 0.193 | 3.70E-16 | 30510241 | Huyghe | 2019 |
| rs55864876 | 11q22.1 | 11 | 100717136 | G | 0.92 | 0.077 | 3.80E-06 | 30510241 | Huyghe | 2019 |
| rs2186607 | 11q22.1 | 11 | 101656397 | T | 0.52 | 0.054 | 1.10E-09 | 30510241 | Huyghe | 2019 |
| rs3087967 | 11q23.1 | 11 | 111156836 | T | 0.29 | 0.112 | 1.90E-31 | 18372901 | Tenesa | 2008 |
| rs35808169 | 12p13.32 | 12 | 4368607 | C | 0.17 | 0.089 | 3.60E-14 | 23263487 | Jia | 2013 |
| rs3217810 | 12p13.32 | 12 | 4388271 | T | 0.13 | 0.118 | 1.60E-15 | 24737748 | Whiffin | 2014 |
| rs3217874 | 12p13.32 | 12 | 4400808 | T | 0.43 | 0.055 | 2.40E-09 | 30510241 | Huyghe | 2019 |
| rs10849433 | 12p13.31 | 12 | 6406904 | C | 0.27 | 0.051 | 2.30E-07 | 30510241 | Huyghe | 2019 |
| rs2250430 | 12p13.31 | 12 | 6421174 | T | 0.71 | 0.060 | 4.10E-09 | 24836286 | Zhang | 2014 |
| rs2710310 | 12p13.2 | 12 | 12035649 | C | 0.76 | 0.015 | 1.60E-01 | 27145994 | Wang | 2016 |
| rs77969132 | 12p11.21 | 12 | 31594813 | T | 0.02 | 0.365 | 4.85E-08 | 30529582 | Lu | 2019 |
| rs11610543 | 12q12 | 12 | 43134191 | G | 0.50 | 0.053 | 1.30E-09 | 30510241 | Huyghe | 2019 |
| rs12372718 | 12q13.12 | 12 | 51171090 | G | 0.39 | 0.090 | 1.90E-23 | 20972440 | Houlston | 2010 |
| rs4759277 | 12q13.3 | 12 | 57533690 | A | 0.35 | 0.053 | 9.40E-09 | 30510241 | Huyghe | 2019 |
| rs597808 | 12q24.12 | 12 | 111973358 | G | 0.52 | 0.074 | 2.60E-16 | 26151821 | Schumacher | 2014 |
| rs1427760 | 12q24.21 | 12 | 115100714 | C | 0.53 | 0.046 | 2.30E-07 | 30510241 | Huyghe | 2019 |
| rs7300312 | 12q24.21 | 12 | 115890922 | C | 0.57 | 0.066 | 8.10E-14 | 29917119 | Schmit | 2019 |
| rs55990915 | 12q24.22 | 12 | 117763309 | A | 0.12 | 0.064 | 1.77E-06 | 26151821 | Schumacher | 2014 |
| rs377429877 | 13q13.2 | 13 | 34092164 | C | 0.61 | 0.047 | 3.40E-07 | 29917119 | Schmit | 2019 |
| rs7333607 | 13q13.3 | 13 | 37462010 | G | 0.24 | 0.076 | 6.30E-13 | 30510241 | Huyghe | 2019 |
| rs45597035 | 13q22.1 | 13 | 73649152 | A | 0.65 | 0.051 | 5.90E-08 | 30510241; 31089142 | Huyghe; Law | 2019 |
| rs78341008 | 13q22.1 | 13 | 73791554 | C | 0.07 | 0.098 | 2.60E-08 | 30510241 | Huyghe | 2019 |
| rs1924816 | 13q22.1 | 13 | 73997961 | A | 0.77 | 0.054 | 2.00E-07 | 30510241 | Huyghe | 2019 |
| rs1330889 | 13q22.3 | 13 | 78609615 | C | 0.87 | 0.104 | 6.50E-10 | 31089142 | Law | 2019 |
| rs8000189 | 13q34 | 13 | 111075881 | T | 0.64 | 0.055 | 1.80E-09 | 30510241 | Huyghe | 2019 |
| rs1951864 | 14q22.2 | 14 | 54369299 | A | 0.37 | 0.041 | 8.80E-06 | 30510241 | Huyghe | 2019 |
| rs35107139 | 14q22.2 | 14 | 54419106 | C | 0.42 | 0.091 | 2.10E-23 | 19011631 | Houlston | 2008 |
| rs4901473 | 14q22.2 | 14 | 54445157 | G | 0.38 | 0.047 | 3.10E-07 | 21655089 | Tomlinson | 2011 |
| rs17094983 | 14q23.1 | 14 | 59189361 | G | 0.88 | 0.069 | 9.90E-07 | 30510241 | Huyghe | 2019 |
| rs8020436 | 14q23.1 | 14 | 59208437 | A | 0.40 | 0.045 | 1.40E-06 | 30510241 | Huyghe | 2019 |
| rs12708491 | 15q13.3 | 15 | 32992836 | G | 0.59 | 0.046 | 1.10E-06 | 21655089 | Tomlinson | 2011 |
| rs2293581 | 15q13.3 | 15 | 33010736 | A | 0.21 | 0.125 | 2.30E-29 | 21655089 | Tomlinson | 2011 |
| rs17816465 | 15q13.3 | 15 | 33156386 | A | 0.21 | 0.071 | 1.50E-10 | 30510241 | Huyghe | 2019 |
| rs12594720 | 15q22.31 | 15 | 67007018 | C | 0.72 | 0.048 | 1.80E-06 | 30510241; 31089142 | Huyghe; Law | 2019 |
| rs56324967 | 15q22.33 | 15 | 67402824 | C | 0.68 | 0.069 | 2.40E-13 | 30510241 | Huyghe | 2019 |
| rs745213 | 15q23 | 15 | 68060389 | G | 0.81 | 0.049 | 8.40E-06 | 30510241 | Huyghe | 2019 |
| rs7495132 | 15q26.1 | 15 | 91172901 | T | 0.12 | 0.104 | 7.92E-10 | 31089142 | Law | 2019 |
| rs9924886 | 16q22.1 | 16 | 68743939 | A | 0.73 | 0.055 | 3.10E-08 | 19011631 | Houlston | 2008 |
| rs9930005 | 16q23.2 | 16 | 80043258 | C | 0.43 | 0.050 | 2.10E-08 | 30510241 | Huyghe | 2019 |
| rs12447408 | 16q24.1 | 16 | 86252544 | A | 0.25 | 0.047 | 4.40E-06 | 30510241 | Huyghe | 2019 |
| rs12149163 | 16q24.1 | 16 | 86339315 | T | 0.50 | 0.049 | 2.80E-08 | 29917119 | Schmit | 2019 |
| rs62042090 | 16q24.1 | 16 | 86703949 | T | 0.22 | 0.048 | 6.60E-06 | 25990418 | Al-Tassan | 2015 |
| rs4968127 | 17p13.3 | 17 | 809643 | G | 0.37 | 0.051 | 6.80E-08 | 24836286 | Zhang | 2014 |
| rs73975586 | 17p13.3 | 17 | 814243 | A | 0.87 | 0.068 | 9.50E-07 | 30510241 | Huyghe | 2019 |
| rs1078643 | 17p12 | 17 | 10707241 | A | 0.76 | 0.075 | 6.60E-12 | 30510241 | Huyghe | 2019 |
| rs983318 | 17q24.3 | 17 | 70413253 | A | 0.25 | 0.060 | 5.60E-09 | 30510241 | Huyghe | 2019 |
| rs373585858 | 17q25.3 | 17 | 80394556 | A | 0.00 | 0.700 | 5.50E-06 | 30510241 | Huyghe | 2019 |
| rs75954926 | 17q25.3 | 17 | 81061048 | G | 0.66 | 0.088 | 2.60E-18 | 30510241 | Huyghe | 2019 |
| rs11874392 | 18q21.1 | 18 | 46453156 | A | 0.55 | 0.161 | 3.80E-74 | 17934461 | Broderick | 2007 |
| rs34797592 | 19p13.11 | 19 | 16417198 | T | 0.12 | 0.087 | 4.20E-10 | 30510241 | Huyghe | 2019 |
| rs28840750 | 19q13.11 | 19 | 33519927 | T | 0.95 | 0.194 | 3.70E-23 | 19011631 | Houlston | 2008 |
| rs1963413 | 19q13.2 | 19 | 41871573 | A | 0.61 | 0.044 | 9.50E-07 | 24836286 | Zhang | 2014 |
| rs12979278 | 19q13.33 | 19 | 49218602 | T | 0.53 | 0.068 | 6.11E-10 | 31089142 | Law | 2019 |
| rs73068325 | 19q13.43 | 19 | 59079096 | T | 0.18 | 0.063 | 4.20E-08 | 30510241 | Huyghe | 2019 |
| rs189583 | 20p12.3 | 20 | 6376457 | G | 0.33 | 0.080 | 1.10E-16 | 19011631 | Houlston | 2008 |
| rs994308 | 20p12.3 | 20 | 6603622 | C | 0.59 | 0.063 | 6.90E-12 | 30510241 | Huyghe | 2019 |
| rs4813802 | 20p12.3 | 20 | 6699595 | G | 0.36 | 0.082 | 2.30E-18 | 21655089 | Tomlinson | 2011 |
| rs28488 | 20p12.3 | 20 | 6762221 | T | 0.64 | 0.071 | 3.30E-14 | 30510241 | Huyghe | 2019 |
| rs11087784 | 20p12.3 | 20 | 7740976 | G | 0.15 | 0.087 | 3.40E-13 | 23263487 | Jia | 2013 |
| rs556532366 | 20p12.3 | 20 | 8568071 | T | 0.00 | 0.476 | 7.00E-06 | 30510241 | Huyghe | 2019 |
| rs6058093 | 20q11.22 | 20 | 33213196 | C | 0.49 | 0.045 | 3.00E-07 | 29917119 | Schmit | 2019 |
| rs6031311 | 20q13.12 | 20 | 42666475 | T | 0.76 | 0.060 | 6.80E-09 | 30510241 | Huyghe | 2019 |
| rs6066825 | 20q13.13 | 20 | 47340117 | A | 0.64 | 0.072 | 5.90E-15 | 26151821 | Schumacher | 2014 |
| rs6067417 | 20q13.13 | 20 | 48983697 | C | 0.56 | 0.045 | 9.40E-07 | 30510241 | Huyghe | 2019 |
| rs6063514 | 20q13.13 | 20 | 49055318 | C | 0.61 | 0.055 | 3.30E-09 | 29917119 | Schmit | 2019 |
| rs6091189 | 20q13.13 | 20 | 49256285 | T | 0.15 | 0.062 | 3.90E-07 | 30510241 | Huyghe | 2019 |
| rs13831 | 20q13.32 | 20 | 57475191 | G | 0.68 | 0.077 | 2.06E-08 | 30529582 | Lu | 2019 |
| rs1741640 | 20q13.33 | 20 | 60932414 | C | 0.77 | 0.115 | 1.10E-26 | 20972440 | Houlston | 2010 |
| rs2738783 | 20q13.33 | 20 | 62308612 | T | 0.20 | 0.059 | 5.30E-08 | 30510241 | Huyghe | 2019 |

SNP, single nucleotide polymorphism; Chr, chromosome; RAF, risk allele frequency.

**Supplementary table 3.** Descriptive characteristics of study participants included in the genome-wide interaction analysis between common variants and menopausal hormone therapy for risk of colorectal cancer

| Study | Case participants | | | | | | |  | Control participants | | | | | |
| --- | --- | --- | --- | --- | --- | --- | --- | --- | --- | --- | --- | --- | --- | --- |
|  | N | No use of MHT n (%) | Any MHT n (%) | E+P n | E-only n | | Age at diagnosis  mean (SD) |  | N | No use of MHT n (%) | Any MHT n (%) | E+P n | E-only n | Age at enrollment  mean (SD) |
| CCFR Set 1 | 259 | 183 (70.7) | 76 (29.3) | 34 | 35 | 58.5 (9.9) | |  | 372 | 206 (55.4) | 166 (44.6) | 65 | 93 | 61.7 (7.9) |
| CCFR Set 3 | 427 | 292 (68.4) | 135 (31.6) | 42 | 68 | 61.8 (7.6) | |  | 250 | 155 (62.0) | 95 (38.0) | 27 | 54 | 62.7 (7.4) |
| CCFR Set 4 | 383 | 259 (67.6) | 124 (32.4) | 41 | 78 | 62.1 (9.3) | |  | 118 | 77 (65.3) | 41 (34.7) | 18 | 16 | 61.7 (9.3) |
| CLUEII | 114 | 98 (86) | 16 (14.0) | - | - | 74.9 (9.5) | |  | 108 | 97 (89.8) | 11 (10.2) | - | - | 65.0 (9.4) |
| Colo 2&3 | 37 | 18 (48.6) | 19 (51.4) | - | - | 66.5 (11.4) | |  | 44 | 17 (38.6) | 27 (61.4) | - | - | 67.3 (9.2) |
| CPSII_1 | 263 | 176 (66.9) | 87 (33.1) | 37 | 50 | 74.8 (5.9) | |  | 255 | 142 (55.7) | 113 (44.3) | 30 | 83 | 74.3 (5.8) |
| CPSII_2 | 172 | 116 (67.4) | 56 (32.6) | 21 | 35 | 79.4 (6.1) | |  | 177 | 101 (57.1) | 76 (42.9) | 30 | 46 | 79.0 (6.0) |
| CRCGEN | 274 | 266 (97.1) | 8 (2.9) | - | - | 68.8 (9.9) | |  | 394 | 377 (95.7) | 17 (4.3) | - | - | 65.9 (9.2) |
| DACHS_1 | 630 | 416 (66.0) | 214 (34.0) | - | - | 71.1 (9.5) | |  | 630 | 294 (46.7) | 336 (53.3) | - | - | 70.4 (8.7) |
| DACHS_2 | 229 | 161 (70.3) | 68 (29.7) | - | - | 72.2 (9.8) | |  | 162 | 88 (54.3) | 74 (45.7) | - | - | 72.3 (9.0) |
| DACHS_3 | 420 | 297 (70.7) | 123 (29.3) | - | - | 71.4 (9.5) | |  | 195 | 113 (57.9) | 82 (42.1) | - | - | 70.3 (10.1) |
| DALS_1 | 267 | 204 (76.4) | 63 (23.6) | - | - | 68.0 (7.7) | |  | 270 | 189 (70.0) | 81 (30.0) | - | - | 67.7 (7.9) |
| DALS_2 | 159 | 127 (79.9) | 32 (20.1) | - | - | 67.5 (7.5) | |  | 194 | 137 (70.6) | 57 (29.4) | - | - | 67.5 (8.2) |
| EPIC | 771 | 544 (70.6) | 227 (29.4) | - | - | 67.2 (6.6) | |  | 865 | 619 (71.6) | 246 (28.4) | - | - | 72.5 (5.9) |
| ESTHER_VERDI | 70 | 52 (74.3) | 18 (25.7) | - | - | 68.4 (6.8) | |  | 70 | 49 (70.0) | 21 (30.0) | - | - | 65.8 (6.7) |
| Kentucky | 397 | 184 (46.3) | 213 (53.7) | 56 | 100 | 64.4 (8.9) | |  | 525 | 150 (28.6) | 375 (71.4) | 86 | 166 | 66.7 (6.6) |
| LCCS | 116 | 90 (77.6) | 26 (22.4) | - | - | 66.1 (6.9) | |  | 108 | 88 (81.5) | 20 (18.5) | - | - | 65.7 (5.5) |
| MCCS_1 | 211 | 159 (75.4) | 52 (24.6) | - | - | 72.0 (7.1) | |  | 184 | 132 (71.7) | 52 (28.3) | - | - | 71.1 (7.2) |
| MCCS_2 | 85 | 65 (76.5) | 20 (23.5) | - | - | 74.3 (8.4) | |  | 86 | 65 (75.6) | 21 (24.4) | - | - | 73.8 (8.0) |
| MEC_1 | 99 | 55 (55.6) | 44 (44.4) | - | 27 | 70.3 (7.9) | |  | 115 | 42 (36.5) | 73 (63.5) | - | 37 | 70.3 (7.6) |
| MEC_2 | 15 | 2 (13.3) | 13 (86.7) | - | 5 | 80.1 (6.2) | |  | 30 | 4 (13.3) | 26 (86.7) | - | 12 | 74.6 (6.1) |
| MECC_3 | 309 | 260 (84.1) | 49 (15.9) | - | - | 69.5 (10.3) | |  | 367 | 290 (79.0) | 77 (21.0) | - | - | 73.0 (10.0) |
| NCCCSII | 219 | 128 (58.4) | 91 (41.6) | - | - | 63.8 (9.8) | |  | 221 | 89 (40.3) | 132 (59.7) | - | - | 65.4 (9.4) |
| NFCCR_2 | 60 | 51 (85.0) | 9 (15.0) | - | - | 61.1 (7.9) | |  | 130 | 104 (80.0) | 26 (20.0) | - | - | 60.2 (7.2) |
| NHS_1_2 | 328 | 174 (53.0) | 154 (47.0) | 7 | 23 | 68.0 (7.4) | |  | 673 | 321 (47.7) | 352 (52.3) | 7 | 42 | 68.5 (6.9) |
| NHS_3_AD | 410 | 187 (45.6) | 223 (54.4) | 10 | 21 | 68.1 (6.7) | |  | 335 | 133 (39.7) | 202 (60.3) | 7 | 15 | 67.9 (6.7) |
| PLCO_1_Rematch | 216 | 125 (57.9) | 91 (42.1) | - | - | 68.8 (6.0) | |  | 123 | 61 (49.6) | 62 (50.4) | - | - | 67.5 (6.2) |
| PLCO_2 | 196 | 110 (56.1) | 86 (43.9) | - | - | 70.6 (6.6) | |  | 163 | 90 (55.2) | 73 (44.8) | - | - | 70.6 (6.3) |
| PLCO_3 | 295 | 157 (53.2) | 138 (46.8) | - | - | 67.0 (7.2) | |  | 1964 | 900 (45.8) | 1064 (54.2) | - | - | 62.1 (5.3) |
| PLCO_4_AD | 434 | 241 (55.5) | 193 (44.5) | - | - | 64.0 (5.9) | |  | 587 | 274 (46.7) | 313 (53.3) | - | - | 61.9 (5.3) |
| REACH_AD | 9 | 7 (77.8) | 2 (22.2) | - | - | 62.9 (4.0) | |  | 75 | 47 (62.7) | 28 (37.3) | - | - | 62.3 (5.6) |
| SMC_COSM | 179 | 90 (50.3) | 89 (49.7) | - | - | 69.7 (9.7) | |  | 330 | 145 (43.9) | 185 (56.1) | - | - | 64.6 (7.8) |
| UKB_1 | 1073 | 996 (92.8) | 77 (7.2) | - | - | 65.4 (5.3) | |  | 4254 | 3928 (92.3) | 326 (7.7) | - | - | 65.4 (5.3) |
| USC_HRT_CRC | 296 | 127 (42.9) | 169 (57.1) | 67 | 75 | 66.3 (5.5) | |  | 400 | 150 (37.5) | 250 (62.5) | 82 | 116 | 65.0 (6.8) |
| VITAL | 114 | 61 (53.5) | 53 (46.5) | - | - | 70.5 (6.4) | |  | 126 | 60 (47.6) | 66 (52.4) | - | - | 71.5 (6.6) |
| WHI_1 | 450 | 297 (66.0) | 153 (34.0) | 58 | 95 | 71.0 (7.1) | |  | 519 | 282 (54.3) | 237 (45.7) | 100 | 137 | 71.2 (7.0) |
| WHI_2 | 977 | 576 (59.0) | 401 (41.0) | 199 | 202 | 72.2 (7.4) | |  | 990 | 512 (51.7) | 478 (48.3) | 217 | 260 | 72.0 (7.2) |
| WHI_3 | 556 | 313 (56.3) | 243 (43.7) | 126 | 117 | 78.6 (6.9) | |  | 558 | 267 (47.8) | 291 (52.2) | 142 | 148 | 78.5 (6.9) |
| Total | 11519 | 7664 (66.5) | 3855 (33.5) | 698 | 931 | - | |  | 16967 | 10795 (63.6) | 6172 (36.4) | 811 | 1225 | - |

N, number; MHT, menopausal hormone therapy; E+P, combined estrogen-progestogen; E-only, estrogen only; SD, standard deviation; CCFR, Colon Cancer Family Registry; CLUEII, Campaign against Cancer and Heart Disease II; Colo 2&3, Hawaii Colorectal Cancer Studies 2 & 3; CPSII, Cancer Prevention Study-II; CRCGEN, Colorectal Cancer Genetics & Genomics; DACHS, Darmkrebs: Chancen der Verhütung durch Screening; DALS, Diet, Activity, and Lifestyle Study; EPIC, European Prospective Investigation into Cancer; ESTHER_VERDI, Epidemiologische Studie zu Chancen der Verhütung, Früherkennung und optimierten THerapie chronischer ERkrankungen in der älteren Bevölkerung; Kentucky, Kentucky Case-Control Study; LCCS, Leeds Colorectal Cancer Study; MCCS, Melbourne Collaborative Cohort Study; MEC, Multiethnic Cohort Study; MECC, Molecular Epidemiology of Colorectal Cancer Study; NCCCSII, The North Carolina Colon Cancer Study II; NFCCR, Newfoundland Case-Control Study; NHS, Nurses’ Health Study; PLCO, Prostate, Lung, Colorectal, and Ovarian Cancer Screening Trial; REACH, Colon Cancer Pathways: Hyperplastic Polyps and Adenomas; SMC_COSM, Swedish Mammography Cohort and Swedish Men Cohort; UKB, UK Biobank; USC_HRT_CRC, University of Southern California Hormone Replacement Therapy Colorectal Cancer Study; VITAL, Cancer Screening Trial VITamins And Lifestyle cohort; WHI, The Women's Health Initiative.

**Supplementary Table 4.** Associations of all MHT variables with colon cancer risk stratified by quartiles of PRS

|  | Quartiles of PRS [OR (95%CI)] | | | | | | | |  |
| --- | --- | --- | --- | --- | --- | --- | --- | --- | --- |
|  |  | PRS.Q1 | PRS.Q2 | PRS.Q3 | PRS.Q4 | Q2 within strata of MHT | Q3 within strata of MHT | Q4 within strata of MHT | |
| No MHT |  | 1 (ref.) | **1.46 (1.30, 1.64)** | **1.95 (1.75, 2.18)** | **2.81 (2.53, 3.13)** | **1.46 (1.30, 1.64)** | **1.95 (1.75, 2.18)** | **2.81 (2.53, 3.13)** | |
| Any MHT |  | **0.83 (0.71, 0.96)** | 1.12 (0.98, 1.29) | **1.44 (1.27, 1.65)** | **1.85 (1.63, 2.10)** | **1.35 (1.16, 1.58)** | **1.74 (1.50, 2.03)** | **2.24 (1.93, 2.59)** | |
| Within strata of PRS.Q |  | **0.83 (0.71, 0.96)** | **0.77 (0.67, 0.87)** | **0.74 (0.66, 0.84)** | **0.66 (0.59, 0.74)** | *P* for multiplicative interaction = 0.077 | | | |
|  |  | RERI | -0.17 (-0.38, 0.04) | **-0.33 (-0.58, -0.09)** | **-0.79^a^ (-1.09, -0.48)** |  | | | |
|  |  |  |  |  |  |  |  |  | |
| No MHT |  | 1 (ref.) | **1.45 (1.20, 1.74)** | **1.86 (1.55, 2.22)** | **2.36 (1.98, 2.82)** | **1.45 (1.20, 1.74)** | **1.86 (1.55, 2.22)** | **2.36 (1.98, 2.82)** | |
| E-only |  | 0.83 (0.64, 1.06) | 0.94 (0.74, 1.20) | 1.26 (1.00, 1.58) | **1.39 (1.12, 1.74)** | 1.15 (0.86, 1.53) | **1.52 (1.15, 2.01)** | **1.69 (1.28, 2.22)** | |
| Within strata of PRS.Q |  | 0.83 (0.64, 1.06) | **0.65 (0.52, 0.83)** | **0.68 (0.55, 0.84)** | **0.59 (0.48, 0.72)** | *P* for multiplicative interaction = 0.249 | | | |
|  |  | RERI | -0.33 (-0.69, 0.04) | **-0.42 (-0.83, -0.01)** | **-0.79 (-1.26, -0.33)** |  | | | |
|  |  |  |  |  |  |  |  |  | |
| No MHT |  | 1 (ref.) | **1.47 (1.22, 1.77)** | **1.89 (1.58, 2.27)** | **2.37 (1.98, 2.83)** | **1.47 (1.22, 1.77)** | **1.89 (1.58, 2.27)** | **2.37 (1.98, 2.83)** | |
| E+P |  | 0.87 (0.65, 1.17) | 1.08 (0.82, 1.42) | **1.51 (1.17, 1.94)** | **1.63 (1.28, 2.09)** | 1.24 (0.88, 1.75) | **1.73 (1.25, 2.41)** | **1.87 (1.35, 2.59)** | |
| Within strata of PRS.Q |  | 0.87 (0.65, 1.17) | **0.73 (0.56, 0.96)** | 0.80 (0.63, 1.02) | **0.69 (0.55, 0.87)** | *P* for multiplicative interaction = 0.617 | | | |
|  |  | RERI | -0.26 (-0.68, 0.16) | -0.25 (-0.73, 0.22) | **-0.61 (-1.13, -0.08)** |  | | | |

PRS.Q, the quartiles of polygenic risk score; OR, odds ratio; 95%CI, 95% confidence interval; MHT, menopausal hormone therapy; E-only, estrogen-only therapy; E+P, combined estrogen-progestogen therapy; RERI, the relative excess risk due to interaction. The regression models were adjusted for age, BMI, study center, and the first three principal components.

The cells in grey present the ORs of colon cancer for MHT users or non-users with different PRS.Q using non-users with PRS.Q1 as reference. ORs of colon cancer for women with different PRS.Q to those with PRS.Q1 stratified by the use of MHT are presented on the right of grey areas. ORs of colon cancer for MHT users to non-users stratified by PRS.Q are presented under the grey areas.

^a^ RERI=-0.79, which equals to OR_11_-OR_10_-OR_01_+1=1.85-2.81-0.83+1, means that the protective effect of MHT use is stronger in the highest quartile of PRS with 79% relative risk reduction more compared to that in the lowest quartile of PRS.

**Supplementary Table 5.** Associations of all MHT variables with rectal cancer risk stratified by quartiles of PRS

|  | Quartiles of PRS [OR (95%CI)] | | | | | | | |  |
| --- | --- | --- | --- | --- | --- | --- | --- | --- | --- |
|  |  | PRS.Q1 | PRS.Q2 | PRS.Q3 | PRS.Q4 | Q2 within strata of MHT | Q3 within strata of MHT | Q4 within strata of MHT | |
| No MHT |  | 1 (ref.) | **1.29 (1.08, 1.55)** | **1.88 (1.59, 2.24)** | **2.85 (2.41, 3.36)** | **1.29 (1.08, 1.55)** | **1.88 (1.59, 2.24)** | **2.85 (2.41, 3.36)** | |
| Any MHT |  | **0.61 (0.47, 0.80)** | 0.91 (0.72, 1.14) | 1.16 (0.93, 1.44) | **1.77 (1.45, 2.16)** | **1.48 (1.11, 1.97)** | **1.90 (1.44, 2.50)** | **2.89 (2.23, 3.76)** | |
| Within strata of PRS.Q |  | **0.61 (0.47, 0.80)** | **0.70 (0.56, 0.88)** | **0.62 (0.50, 0.75)** | **0.62 (0.52, 0.74)** | *P* for multiplicative interaction = 0.802 | | | |
|  |  | RERI | 0 (-0.30, 0.30) | -0.33 (-0.69, 0.02) | **-0.69^a^ (-1.14, -0.24)** |  | | | |
|  |  |  |  |  |  |  |  |  | |
| No MHT |  | 1 (ref.) | **1.15 (0.83, 1.59)** | **1.77 (1.31, 2.40)** | **2.36 (1.76, 3.16)** | **1.15 (0.83, 1.59)** | **1.77 (1.31, 2.40)** | **2.36 (1.76, 3.16)** | |
| E-only |  | 0.72 (0.45, 1.16) | 0.76 (0.48, 1.20) | 1.03 (0.68, 1.57) | 1.25 (0.84, 1.84) | 1.05 (0.59, 1.86) | 1.43 (0.84, 2.45) | **1.73 (1.03, 2.90)** | |
| Within strata of PRS.Q |  | 0.72 (0.45, 1.16) | 0.66 (0.42, 1.04) | **0.58 (0.39, 0.86)** | **0.53 (0.37, 0.75)** | *P* for multiplicative interaction = 0.741 | | | |
|  |  | RERI | -0.11 (-0.67, 0.45) | -0.46 (-1.13, 0.21) | **-0.83 (-1.6, -0.06)** |  | | | |
|  |  |  |  |  |  |  |  |  | |
| No MHT |  | 1 (ref.) | 1.17 (0.84, 1.63) | **1.77 (1.31, 2.41)** | **2.37 (1.76, 3.19)** | 1.17 (0.84, 1.63) | **1.77 (1.31, 2.41)** | **2.37 (1.76, 3.19)** | |
| E+P |  | **0.52 (0.28, 0.98)** | 0.92 (0.56, 1.52) | 0.82 (0.50, 1.35) | 1.40 (0.91, 2.15) | 1.76 (0.86, 3.61) | **1.57 (0.76, 3.21)** | **2.68 (1.36, 5.25)** | |
| Within strata of PRS.Q |  | **0.52 (0.28, 0.98)** | 0.79 (0.48, 1.29) | **0.46 (0.29, 0.74)** | **0.59 (0.40, 0.88)** | *P* for multiplicative interaction = 0.470 | | | |
|  |  | RERI | 0.23 (-0.36, 0.83) | -0.48 (-1.15, 0.2) | -0.49 (-1.29, 0.31) |  | | | |

PRS.Q, the quartiles of polygenic risk score; OR, odds ratio; 95%CI, 95% confidence interval; MHT, menopausal hormone therapy; E-only, estrogen-only therapy; E+P, combined estrogen-progestogen therapy; RERI, the relative excess risk due to interaction. The regression models were adjusted for age, BMI, study center, and the first three principal components.

The cells in grey present the ORs of rectal cancer for MHT users or non-users with different PRS.Q using non-users with PRS.Q1 as reference. ORs of rectal cancer for women with different PRS.Q to those with PRS.Q1 stratified by the use of MHT are presented on the right of grey areas. ORs of rectal cancer for MHT users to non-users stratified by PRS.Q are presented under the grey areas.

^a^ RERI=-0.69, which equals to OR_11_-OR_10_-OR_01_+1=1.77-2.85-0.61+1, means that the protective effect of MHT use is stronger in the highest quartile of PRS with 69% relative risk reduction more compared to that in the lowest quartile of PRS.

**Supplementary Table 6.** Associations of all MHT variables with proximal colon cancer risk stratified by quartiles of PRS

|  | Quartiles of PRS [OR (95%CI)] | | | | | | | |  |
| --- | --- | --- | --- | --- | --- | --- | --- | --- | --- |
|  |  | PRS.Q1 | PRS.Q2 | PRS.Q3 | PRS.Q4 | Q2 within strata of MHT | Q3 within strata of MHT | Q4 within strata of MHT | |
| No MHT |  | 1 (ref.) | **1.34 (1.17, 1.54)** | **1.80 (1.57, 2.05)** | **2.44 (2.14, 2.77)** | **1.34 (1.17, 1.54)** | **1.80 (1.57, 2.05)** | **2.44 (2.14, 2.77)** | |
| Any MHT |  | **0.79 (0.67, 0.95)** | 1.08 (0.92, 1.27) | **1.36 (1.16, 1.59)** | **1.72 (1.48, 1.99)** | **1.36 (1.13, 1.64)** | **1.71 (1.43, 2.05)** | **2.16 (1.81, 2.58)** | |
| Within strata of PRS.Q |  | **0.79 (0.67, 0.95)** | **0.81 (0.69, 0.94)** | **0.76 (0.65, 0.87)** | **0.71 (0.62, 0.81)** | *P* for multiplicative interaction = 0.558 | | | |
|  |  | RERI | -0.05 (-0.29, 0.19) | -0.23 (-0.51, 0.04) | **-0.51^a^ (-0.84, -0.19)** |  | | | |
|  |  |  |  |  |  |  |  |  | |
| No MHT |  | 1 (ref.) | **1.31 (1.06, 1.61)** | **1.75 (1.43, 2.14)** | **1.99 (1.64, 2.43)** | **1.31 (1.06, 1.61)** | **1.75 (1.43, 2.14)** | **1.99 (1.64, 2.43)** | |
| E-only |  | 0.68 (0.50, 0.92) | 0.95 (0.72, 1.24) | 1.15 (0.89, 1.49) | **1.33 (1.04, 1.71)** | 1.40 (1.00, 1.96) | **1.70 (1.22, 2.37)** | **1.97 (1.42, 2.72)** | |
| Within strata of PRS.Q |  | 0.68 (0.50, 0.92) | **0.72 (0.56, 0.94)** | **0.66 (0.52, 0.84)** | **0.67 (0.53, 0.84)** | *P* for multiplicative interaction = 0.959 | | | |
|  |  | RERI | -0.04 (-0.40, 0.33) | -0.27 (-0.69, 0.15) | -0.34 (-0.79, 0.11) |  | | | |
|  |  |  |  |  |  |  |  |  | |
| No MHT |  | 1 (ref.) | **1.32 (1.07, 1.63)** | **1.78 (1.45, 2.18)** | **1.98 (1.62, 2.42)** | **1.32 (1.07, 1.63)** | **1.78 (1.45, 2.18)** | **1.98 (1.62, 2.42)** | |
| E+P |  | 0.82 (0.59, 1.15) | 1.05 (0.77, 1.42) | **1.40 (1.05, 1.86)** | **1.51 (1.14, 1.99)** | 1.27 (0.86, 1.89) | **1.70 (1.17, 2.48)** | **1.83 (1.27, 2.66)** | |
| Within strata of PRS.Q |  | 0.82 (0.59, 1.15) | 0.79 (0.58, 1.07) | 0.79 (0.60, 1.03) | **0.76 (0.58, 0.99)** | *P* for multiplicative interaction = 0.988 | | | |
|  |  | RERI | -0.10 (-0.54, 0.35) | -0.20 (-0.70, 0.30) | -0.30 (-0.82, 0.23) |  | | | |

PRS.Q, the quartiles of polygenic risk score; OR, odds ratio; 95%CI, 95% confidence interval; MHT, menopausal hormone therapy; E-only, estrogen-only therapy; E+P, combined estrogen-progestogen therapy; RERI, the relative excess risk due to interaction. The regression models were adjusted for age, BMI, study center, and the first three principal components.

The cells in grey present the ORs of proximal colon cancer for MHT users or non-users with different PRS.Q using non-users with PRS.Q1 as reference. ORs of proximal colon cancer for women with different PRS.Q to those with PRS.Q1 stratified by the use of MHT are presented on the right of grey areas. ORs of proximal colon cancer for MHT users to non-users stratified by PRS.Q are presented under the grey areas.

^a^ RERI=-0.51, which equals to OR_11_-OR_10_-OR_01_+1=1.72-2.44-0.79+1, means that the protective effect of MHT use is stronger in the highest quartile of PRS with 51% relative risk reduction more compared to that in the lowest quartile of PRS.

**Supplementary Table 7.** Associations of all MHT variables with distal colon cancer risk stratified by quartiles of PRS

|  | Quartiles of PRS [OR (95%CI)] | | | | | | | |  |
| --- | --- | --- | --- | --- | --- | --- | --- | --- | --- |
|  |  | PRS.Q1 | PRS.Q2 | PRS.Q3 | PRS.Q4 | Q2 within strata of MHT | Q3 within strata of MHT | Q4 within strata of MHT | |
| No MHT |  | 1 (ref.) | **1.65 (1.39, 1.96)** | **2.17 (1.84, 2.56)** | **3.32 (2.84, 3.90)** | **1.65 (1.39, 1.96)** | **2.17 (1.84, 2.56)** | **3.32 (2.84, 3.90)** | |
| Any MHT |  | 0.85 (0.68, 1.07) | 1.14 (0.93, 1.41) | **1.53 (1.25, 1.86)** | **2.06 (1.71, 2.48)** | **1.34 (1.06, 1.71)** | **1.80 (1.43, 2.26)** | **2.42 (1.94, 3.02)** | |
| Within strata of PRS.Q |  | 0.85 (0.68, 1.07) | **0.69 (0.57, 0.84)** | **0.71 (0.59, 0.84)** | **0.62 (0.53, 0.72)** | *P* for multiplicative interaction = 0.149 | | | |
|  |  | RERI | **-0.35 (-0.70, -0.01)** | **-0.49 (-0.88, -0.09)** | **-1.12^a^ (-1.62, -0.62)** |  | | | |
|  |  |  |  |  |  |  |  |  | |
| No MHT |  | 1 (ref.) | **1.75 (1.30, 2.36)** | **2.04 (1.52, 2.73)** | **3.26 (2.47, 4.30)** | **1.75 (1.30, 2.36)** | **2.04 (1.52, 2.73)** | **3.26 (2.47, 4.30)** | |
| E-only |  | 1.12 (0.75, 1.67) | 0.99 (0.66, 1.47) | **1.52 (1.07, 2.18)** | **1.63 (1.15, 2.31)** | 0.88 (0.55, 1.39) | 1.36 (0.89, 2.06) | 1.45 (0.96, 2.19) | |
| Within strata of PRS.Q |  | 1.12 (0.75, 1.67) | **0.56 (0.39, 0.82)** | 0.75 (0.54, 1.03) | **0.50 (0.37, 0.67)** | *P* for multiplicative interaction = **0.010** | | | |
|  |  | RERI | **-0.89 (-1.62, -0.16)** | -0.64 (-1.39, 0.12) | **-1.75 (-2.73, -0.78)** |  | | | |
|  |  |  |  |  |  |  |  |  | |
| No MHT |  | 1 (ref.) | **1.83 (1.35, 2.48)** | **2.10 (1.56, 2.83)** | **3.41 (2.57, 4.53)** | **1.83 (1.35, 2.48)** | **2.10 (1.56, 2.83)** | **3.41 (2.57, 4.53)** | |
| E+P |  | 1.05 (0.66, 1.67) | 1.13 (0.72, 1.77) | **1.80 (1.22, 2.66)** | **2.02 (1.39, 2.96)** | 1.08 (0.62, 1.87) | **1.72 (1.04, 2.84)** | **1.94 (1.18, 3.17)** | |
| Within strata of PRS.Q |  | 1.05 (0.66, 1.67) | **0.61 (0.40, 0.94)** | 0.86 (0.60, 1.22) | **0.59 (0.43, 0.83)** | *P* for multiplicative interaction = 0.155 | | | |
|  |  | RERI | -0.75 (-1.56, 0.05) | -0.34 (-1.19, 0.50) | **-1.43 (-2.50, -0.37)** |  | | | |

PRS.Q, the quartiles of polygenic risk score; OR, odds ratio; 95%CI, 95% confidence interval; MHT, menopausal hormone therapy; E-only, estrogen-only therapy; E+P, combined estrogen-progestogen therapy; RERI, the relative excess risk due to interaction. The regression models were adjusted for age, BMI, study center, and the first three principal components.

The cells in grey present the ORs of distal colon cancer for MHT users or non-users with different PRS.Q using non-users with PRS.Q1 as reference. ORs of distal colon cancer for women with different PRS.Q to those with PRS.Q1 stratified by the use of MHT are presented on the right of grey areas. ORs of distal colon cancer for MHT users to non-users stratified by PRS.Q are presented under the grey areas.

^a^ RERI=-1.12, which equals to OR_11_-OR_10_-OR_01_+1=2.06-3.32-0.85+1, means that the protective effect of MHT use is stronger in the highest quartile of PRS with 112% relative risk reduction more compared to that in the lowest quartile of PRS.

**Supplementary Table 8.** 30-year cumulative risk estimates (%) of colon cancer for 50-year-old women by use of all MHT variables and quartiles of PRS

|  | 30-year absolute risk, % (95% CI) | | | | | | | | | | |
| --- | --- | --- | --- | --- | --- | --- | --- | --- | --- | --- | --- |
|  | Ca/Co, n | PRS.Q1 |  | Ca/Co, n | PRS.Q2 |  | Ca/Co, n | PRS.Q3 |  | Ca/Co, n | PRS.Q4 |
| No MHT | 749/2693 | 1.63 (1.46, 1.80) |  | 1103/2744 | 2.49 (2.27, 2.71) |  | 1387/2676 | 3.16 (2.90, 3.41) |  | 1990/2680 | 4.62 (4.29, 4.94) |
| Any MHT | 397/1544 | 1.37 (1.17, 1.58) |  | 554/1541 | 1.76 (1.52, 2.01) |  | 681/1470 | 2.22 (1.88, 2.56) |  | 929/1614 | 2.83 (2.55, 3.11) |
| *Diff* |  | *0.26* |  |  | *0.73* |  |  | *0.94* |  |  | *1.79* |
| *P* value |  | 9.28x10^-2^ |  |  | 3.36x10^-5^ |  |  | 1.93x10^-5^ |  |  | 1.11x10^-15^ |
|  |  |  |  |  |  |  |  |  |  |  |  |
| No MHT | 316/628 | 1.84 (1.52, 2.16) |  | 479/661 | 2.44 (2.09, 2.80) |  | 545/593 | 3.22 (2.87, 3.58) |  | 772/658 | 4.16 (3.74, 4.58) |
| E-Only | 124/279 | 1.46 (1.09, 1.84) |  | 156/317 | 1.61 (1.19, 2.04) |  | 201/285 | 2.41 (1.79, 3.02) |  | 246/344 | 2.54 (1.92, 3.16) |
| *Diff* |  | *0.38* |  |  | *0.83* |  |  | *0.81* |  |  | *1.62* |
| *P* value |  | 0.21 |  |  | 3.24x10^-3^ |  |  | 4.18x10^-2^ |  |  | 9.29x10^-6^ |
|  |  |  |  |  |  |  |  |  |  |  |  |
| No MHT | 308/618 | 1.77 (1.56, 1.99) |  | 473/649 | 2.26 (1.87, 2.64) |  | 535/583 | 3.10 (2.69, 3.51) |  | 756/644 | 3.86 (3.49, 4.22) |
| E+P | 83/188 | 1.71 (0.83, 2.60) |  | 121/207 | 1.84 (1.01, 2.68) |  | 155/193 | 2.36 (1.62, 3.10) |  | 197/223 | 2.99 (1.69, 4.28) |
| *Diff* |  | *0.06* |  |  | *0.42* |  |  | *0.74* |  |  | *0.87* |
| *P* value |  | 0.85 |  |  | 0.51 |  |  | 5.98x10^-2^ |  |  | 0.29 |

Ca/Co, number of cases patients and controls individuals; PRS.Q, the quartiles of polygenic risk score; 95% CI, 95% confidence interval; MHT, menopausal hormone therapy; E-only, estrogen-only therapy; E+P, combined estrogen-progestogen therapy; *Diff*, the estimated difference of absolute risks between MHT users and non-users; *P* value, an alpha level of 0.05 (two-sided) was considered to be significant for comparing the absolute risks of MHT users to those of non-users.

**Supplementary Table 9.** 30-year cumulative risk estimates (%) of rectal cancer for 50-year-old women by use of all MHT variables and quartiles of PRS

|  | 30-year absolute risk, % (95% CI) | | | | | | | | | | |
| --- | --- | --- | --- | --- | --- | --- | --- | --- | --- | --- | --- |
|  | Ca/Co, n | PRS.Q1 |  | Ca/Co, n | PRS.Q2 |  | Ca/Co, n | PRS.Q3 |  | Ca/Co, n | PRS.Q4 |
| No MHT | 246/2693 | 0.60 (0.52, 0.68) |  | 317/2744 | 0.82 (0.69, 0.94) |  | 443/2676 | 1.12 (0.96, 1.27) |  | 661/2680 | 1.71 (1.53, 1.90) |
| Any MHT | 89/1544 | 0.34 (0.24, 0.43) |  | 137/1541 | 0.57 (0.43, 0.71) |  | 170/1470 | 0.68 (0.54, 0.83) |  | 268/1614 | 1.02 (0.84, 1.19) |
| *Diff* |  | *0.26* |  |  | *0.25* |  |  | *0.44* |  |  | *0.69* |
| *P* value |  | 9.80x10^-5^ |  |  | 2.07x10^-2^ |  |  | 2.21x10^-5^ |  |  | 1.95x10^-8^ |
|  |  |  |  |  |  |  |  |  |  |  |  |
| No MHT | 83/628 | 0.69 (0.49, 0.88) |  | 96/661 | 0.72 (0.52, 0.93) |  | 144/593 | 1.18 (0.94, 1.43) |  | 208/658 | 1.44 (1.20, 1.68) |
| E-Only | 25/279 | 0.60 (0.31, 0.88) |  | 30/317 | 0.51 (0.25, 0.76) |  | 40/285 | 0.73 (0.37, 1.08) |  | 53/344 | 0.74 (0.44, 1.04) |
| *Diff* |  | *0.09* |  |  | *0.21* |  |  | *0.45* |  |  | *0.70* |
| *P* value |  | 0.49 |  |  | 0.20 |  |  | 3.94x10^-2^ |  |  | 4.68x10^-4^ |
|  |  |  |  |  |  |  |  |  |  |  |  |
| No MHT | 82/618 | 0.67 (0.52, 0.82) |  | 94/649 | 0.65 (0.48, 0.82) |  | 139/583 | 1.18 (0.91, 1.44) |  | 200/644 | 1.38 (1.13, 1.62) |
| E+P | 13/188 | 0.21 (-0.02, 0.44) |  | 25/207 | 0.58 (0.13, 1.03) |  | 23/193 | 0.41 (0.17, 0.65) |  | 43/223 | 1.36 (0.33, 2.39) |
| *Diff* |  | *0.46* |  |  | *0.07* |  |  | *0.77* |  |  | *0.02* |
| *P* value |  | 1.38x10^-3^ |  |  | 0.81 |  |  | 1.90x10^-5^ |  |  | 0.92 |

Ca/Co, number of cases patients and controls individuals; PRS.Q, the quartiles of polygenic risk score; 95% CI, 95% confidence interval; MHT, menopausal hormone therapy; E-only, estrogen-only therapy; E+P, combined estrogen-progestogen therapy; *Diff*, the estimated difference of absolute risks between MHT users and non-users; *P* value, an alpha level of 0.05 (two-sided) was considered to be significant for comparing the absolute risks of MHT users to those of non-users.

**Supplementary Table 10.** 30-year cumulative risk estimates (%) of proximal colon cancer for 50-year-old women by use of all MHT variables and quartiles of PRS

|  | 30-year absolute risk, % (95% CI) | | | | | | | | | | |
| --- | --- | --- | --- | --- | --- | --- | --- | --- | --- | --- | --- |
|  | Ca/Co, n | PRS.Q1 |  | Ca/Co, n | PRS.Q2 |  | Ca/Co, n | PRS.Q3 |  | Ca/Co, n | PRS.Q4 |
| No MHT | 479/2693 | 1.04 (0.90, 1.17) |  | 650/2744 | 1.49 (1.35, 1.62) |  | 800/2676 | 1.91 (1.71, 2.10) |  | 1090/2680 | 2.61 (2.39, 2.82) |
| Any MHT | 247/1544 | 0.80 (0.64, 0.96) |  | 352/1541 | 1.08 (0.90, 1.26) |  | 420/1470 | 1.36 (1.13, 1.58) |  | 566/1614 | 1.75 (1.47, 2.03) |
| *Diff* |  | *0.24* |  |  | *0.41* |  |  | *0.55* |  |  | *0.86* |
| *P* value |  | 2.25x10^-2^ |  |  | 7.76x10^-4^ |  |  | 6.63x10^-5^ |  |  | 2.02x10^-5^ |
|  |  |  |  |  |  |  |  |  |  |  |  |
| No MHT | 232/628 | 1.16 (0.94, 1.38) |  | 313/661 | 1.43 (1.19, 1.67) |  | 366/593 | 2.07 (1.75, 2.40) |  | 466/658 | 2.31 (2.04, 2.59) |
| E-Only | 74/279 | 0.67 (0.46, 0.88) |  | 111/317 | 0.96 (0.72, 1.21) |  | 134/285 | 1.39 (0.94, 1.84) |  | 163/344 | 1.73 (1.35, 2.11) |
| *Diff* |  | *0.49* |  |  | *0.47* |  |  | *0.68* |  |  | *0.58* |
| *P* value |  | 1.13x10^-3^ |  |  | 6.25x10^-3^ |  |  | 1.12x10^-2^ |  |  | 1.27x10^-2^ |
|  |  |  |  |  |  |  |  |  |  |  |  |
| No MHT | 227/618 | 1.10 (0.92, 1.28) |  | 310/649 | 1.33 (1.07, 1.60) |  | 360/583 | 1.96 (1.68, 2.23) |  | 454/644 | 2.13 (1.88, 2.39) |
| E+P | 55/188 | 0.99 (0.42, 1.55) |  | 84/207 | 1.26 (0.53, 1.99) |  | 103/193 | 1.56 (1.07, 2.06) |  | 129/223 | 1.76 (0.97, 2.55) |
| *Diff* |  | *0.11* |  |  | *0.07* |  |  | *0.40* |  |  | *0.37* |
| *P* value |  | 0.89 |  |  | 0.83 |  |  | 9.47x10^-2^ |  |  | 0.51 |

Ca/Co, number of cases patients and controls individuals; PRS.Q, the quartiles of polygenic risk score; 95% CI, 95% confidence interval; MHT, menopausal hormone therapy; E-only, estrogen-only therapy; E+P, combined estrogen-progestogen therapy; *Diff*, the estimated difference of absolute risks between MHT users and non-users; *P* value, an alpha level of 0.05 (two-sided) was considered to be significant for comparing the absolute risks of MHT users to those of non-users.

**Supplementary Table 11.** 30-year cumulative risk estimates (%) of distal colon cancer for 50-year-old women by use of all MHT variables and quartiles of PRS

|  | 30-year absolute risk, % (95% CI) | | | | | | | | | | |
| --- | --- | --- | --- | --- | --- | --- | --- | --- | --- | --- | --- |
|  | Ca/Co, n | PRS.Q1 |  | Ca/Co, n | PRS.Q2 |  | Ca/Co, n | PRS.Q3 |  | Ca/Co, n | PRS.Q4 |
| No MHT | 249/2693 | 0.51 (0.44, 0.59) |  | 411/2744 | 0.87 (0.76, 0.99) |  | 533/2676 | 1.08 (0.97, 1.19) |  | 806/2680 | 1.78 (1.64, 1.92) |
| Any MHT | 134/1544 | 0.47 (0.35, 0.59) |  | 188/1541 | 0.61 (0.48, 0.73) |  | 244/1470 | 0.74 (0.62, 0.86) |  | 345/1614 | 0.98 (0.84, 1.12) |
| *Diff* |  | *0.04* |  |  | *0.26* |  |  | *0.34* |  |  | *0.80* |
| *P* value |  | 0.51 |  |  | 1.39x10^-3^ |  |  | 1.56x10^-5^ |  |  | 3.60x10^-16^ |
|  |  |  |  |  |  |  |  |  |  |  |  |
| No MHT | 78/628 | 0.57 (0.41, 0.74) |  | 150/661 | 0.87 (0.69, 1.05) |  | 161/593 | 0.94 (0.77, 1.11) |  | 279/658 | 1.70 (1.45, 1.96) |
| E-Only | 42/279 | 0.71 (0.44, 0.98) |  | 44/317 | 0.58 (0.34, 0.83) |  | 63/285 | 0.88 (0.53, 1.24) |  | 79/344 | 0.71 (0.48, 0.94) |
| *Diff* |  | *-0.14* |  |  | *0.29* |  |  | *0.06* |  |  | *0.99* |
| *P* value |  | 0.30 |  |  | 6.58x10^-2^ |  |  | 0.68 |  |  | 5.21x10^-9^ |
|  |  |  |  |  |  |  |  |  |  |  |  |
| No MHT | 75/618 | 0.58 (0.44, 0.71) |  | 147/649 | 0.80 (0.63, 0.96) |  | 157/583 | 0.93 (0.79, 1.08) |  | 275/644 | 1.61 (1.36, 1.87) |
| E+P | 27/188 | 0.66 (0.12, 1.21) |  | 34/207 | 0.44 (0.15, 0.74) |  | 51/193 | 0.67 (0.33, 1.00) |  | 66/223 | 1.13 (0.59, 1.66) |
| *Diff* |  | *-0.08* |  |  | *0.36* |  |  | *0.26* |  |  | *0.48* |
| *P* value |  | 0.55 |  |  | 3.86x10^-2^ |  |  | 0.30 |  |  | 9.67x10^-2^ |

Ca/Co, number of cases patients and controls individuals; PRS.Q, the quartiles of polygenic risk score; 95% CI, 95% confidence interval; MHT, menopausal hormone therapy; E-only, estrogen-only therapy; E+P, combined estrogen-progestogen therapy; *Diff*, the estimated difference of absolute risks between MHT users and non-users; *P* value, an alpha level of 0.05 (two-sided) was considered to be significant for comparing the absolute risks of MHT users to those of non-users.


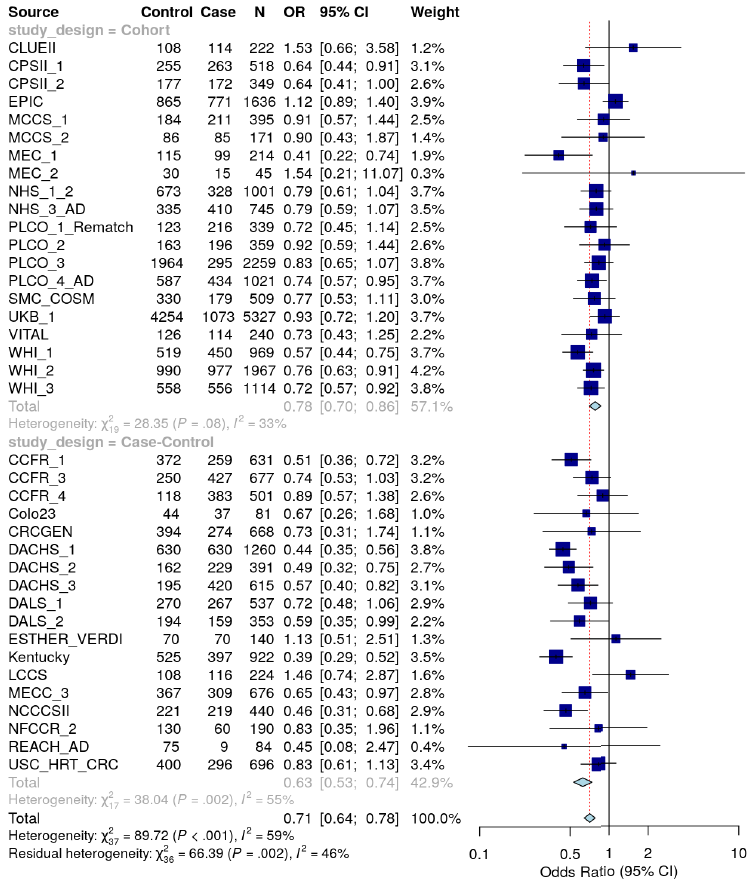


**Supplementary Figure 1.** Association of any menopausal hormone therapy use with the risk of colorectal cancer


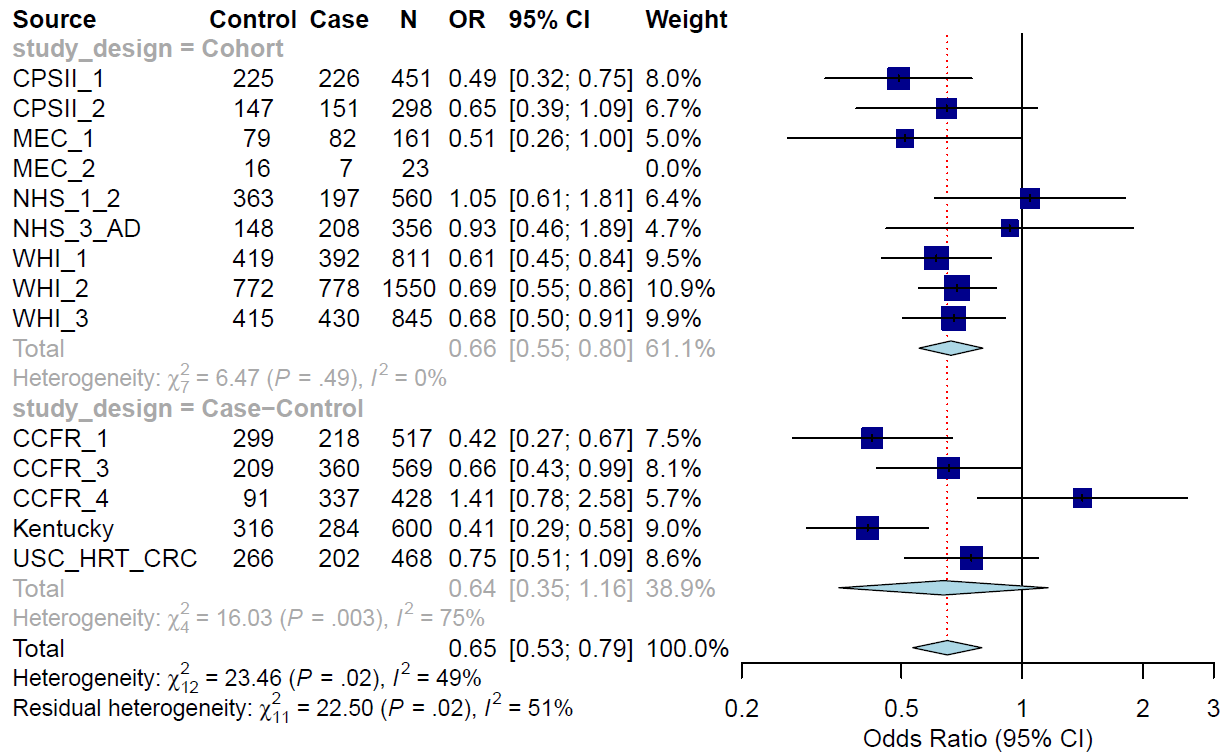


**Supplementary Figure 2**. Association of the use of estrogen-only with the risk of colorectal cancer


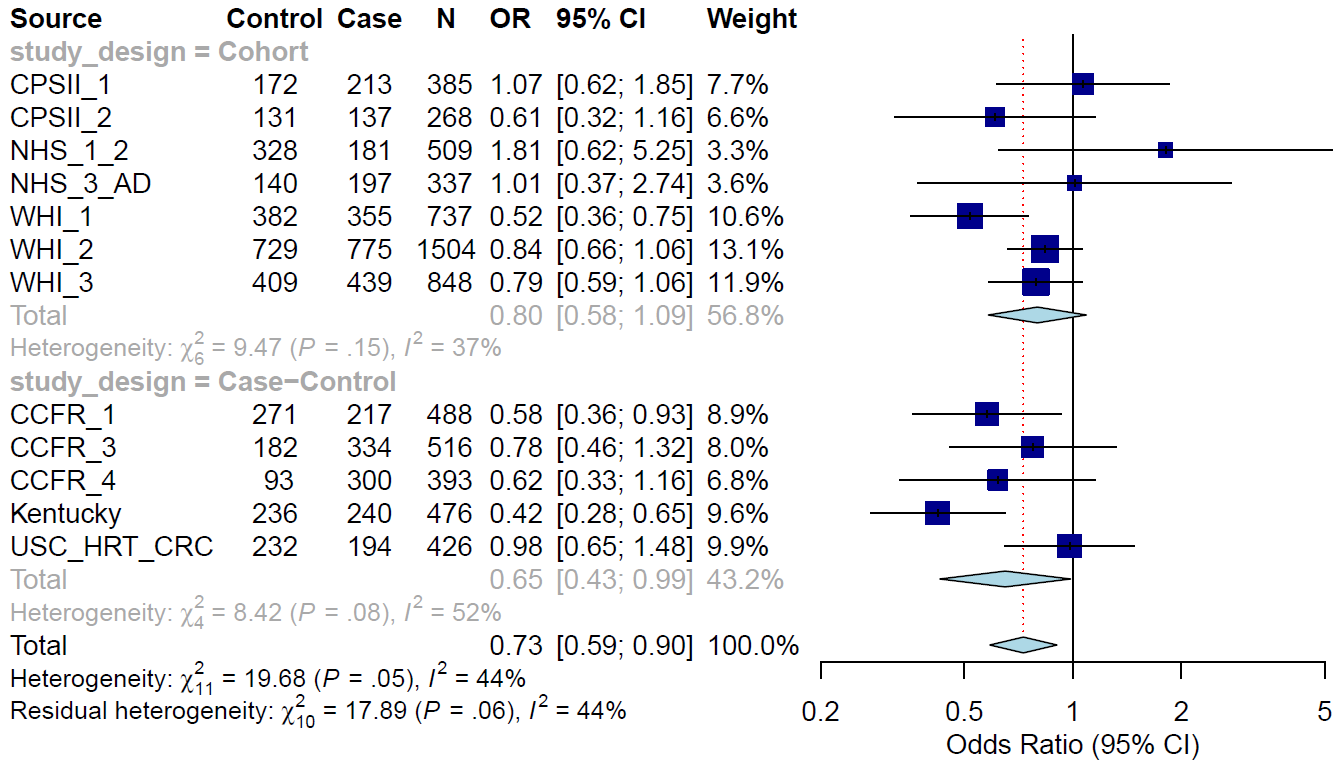


**Supplementary Figure 3**. Association of the use of combined estrogen-progestogen with the risk of colorectal cancer

**Supplementary Figure 4**. Effects of polygenic risk score and the use of estrogen-only on colorectal cancer risk. PRS.Q, the quartiles of polygenic risk score; OR, odds ratio; 95%CI, 95% confidence interval; CRC, colorectal cancer; MHT, menopausal hormone therapy; E-only, estrogen-only therapy; RERI, the relative excess risk due to interaction. The regression model was adjusted for age, BMI, study center, and the first three principal components.


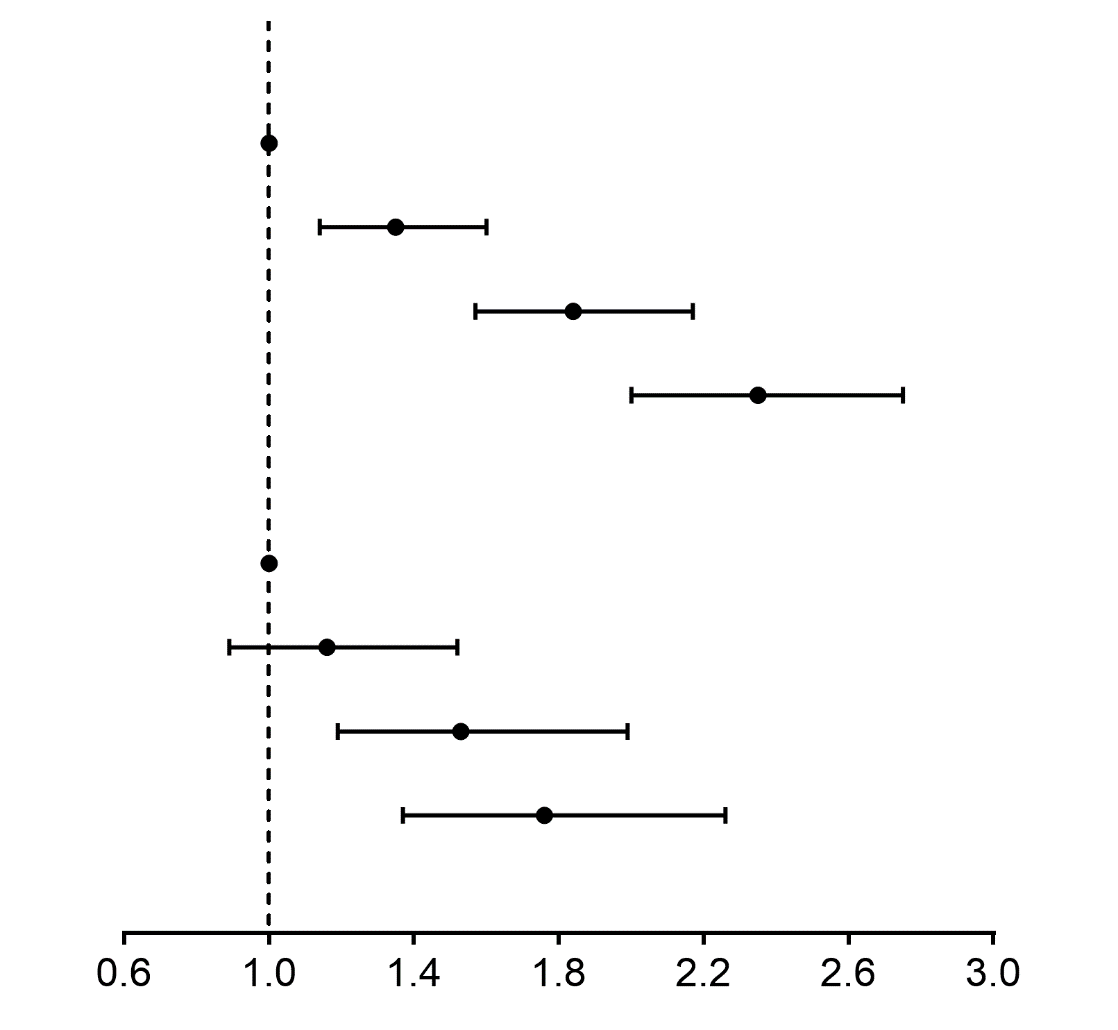


No MHT

Q1

Q2

Q3

Q4

*P* for trend

E-only use

Q1

Q2

Q3

Q4

*P* for trend

1 (Reference)

1.35 (1.14, 1.60)

1.84 (1.57, 2.17)

2.35 (2.00, 2.75)

0.003

1 (Reference)

1.16 (0.89, 1.52)

1.53 (1.19, 1.99)

1.76 (1.37, 2.26)

0.010

**PRS quartile**

**N of Cases/Controls**

**OR for CRC**

**OR (95%CI)**

**RERI (95%CI)**

-0.22 (-0.53, 0.09)

-0.43 (-0.79, -0.06)

-0.76 (-1.17, -0.34)

*P* for multiplicative interaction = 0.302

322 / 344

253 / 285

199 / 317

157 / 279

1,088 / 658

776 / 593

630 / 661

447 / 628


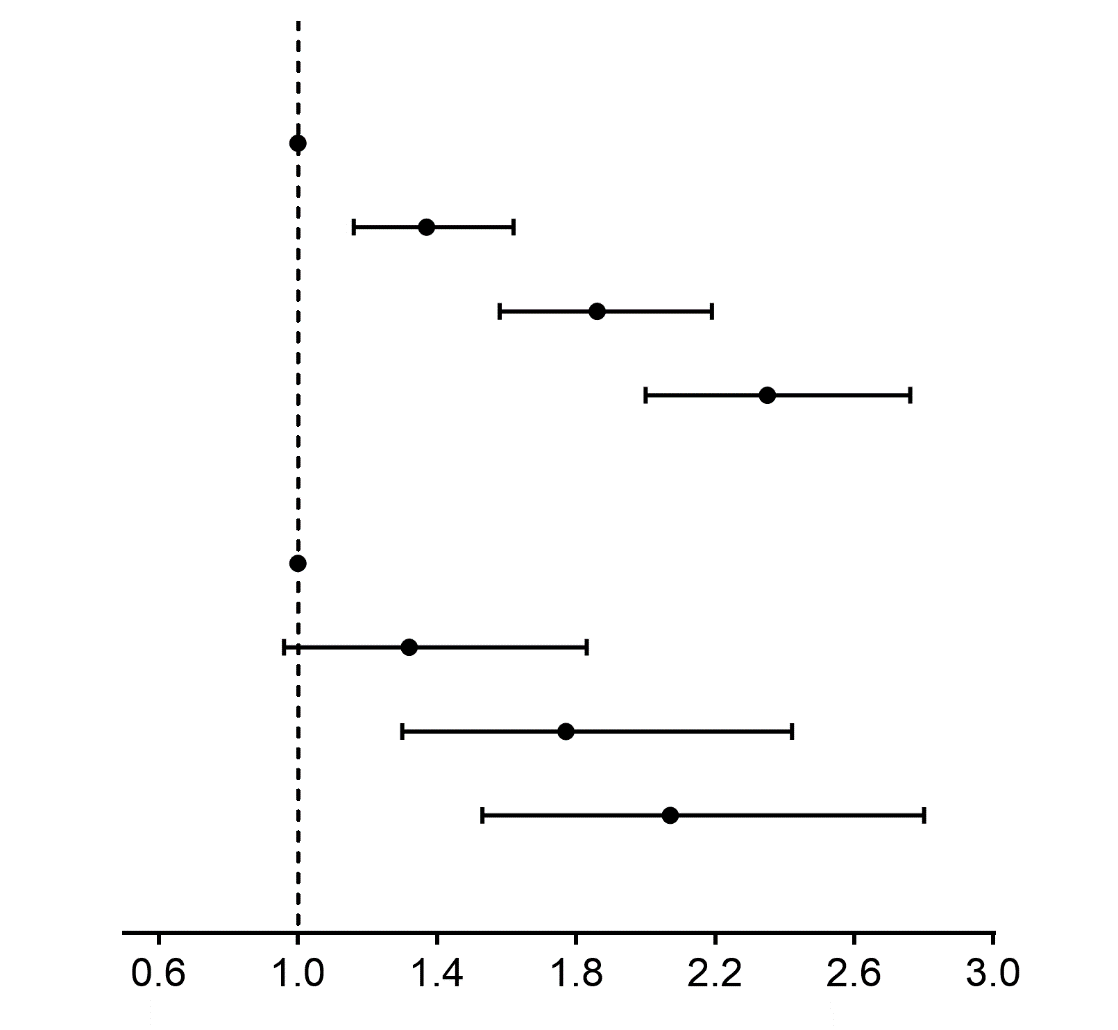


No MHT

Q1

Q2

Q3

Q4

*P* for trend

E+P use

Q1

Q2

Q3

Q4

*P* for trend

1 (Reference)

1.37 (1.16, 1.62)

1.86 (1.58, 2.19)

2.35 (2.00, 2.76)

0.002

1 (Reference)

1.32 (0.96, 1.83)

1.77 (1.30, 2.42)

2.07 (1.53, 2.80)

0.003

**PRS quartile**

**OR for CRC**

**OR (95%CI)**

**RERI (95%CI)**

-0.12 (-0.48, 0.23)

-0.27 (-0.68, 0.15)

-0.53 (-1.00, -0.07)

*P* for multiplicative interaction = 0.886

**Supplementary Figure 5**. Effects of polygenic risk score and the use of combined estrogen-progestogen therapy on colorectal cancer risk. PRS.Q, the quartiles of polygenic risk score; OR, odds ratio; 95%CI, 95% confidence interval; CRC, colorectal cancer; MHT, menopausal hormone therapy; E+P, combined estrogen-progestogen therapy; RERI, the relative excess risk due to interaction. The regression model was adjusted for age, BMI, study center, and the first three principal components.

**N of Cases/Controls**

256 / 223

187 / 193

155 / 207

100 / 188

1,064 / 644

760 / 583

622 / 649

438 / 618

**
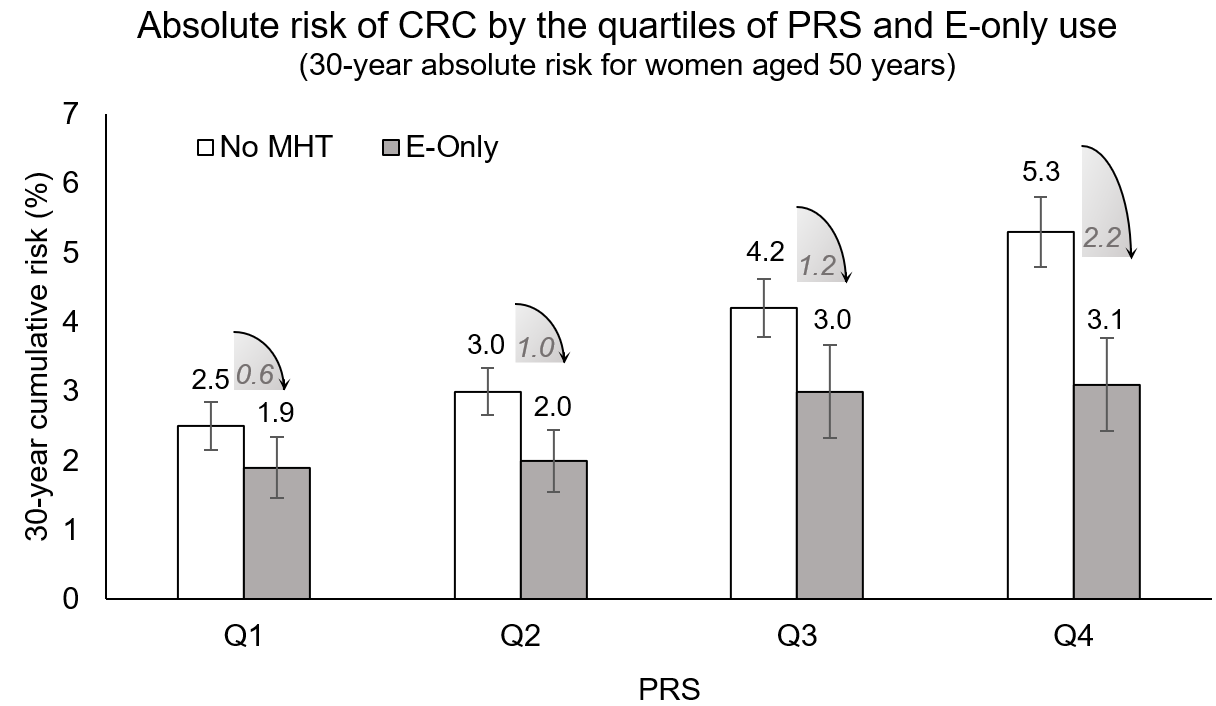
**

**Supplementary Figure 6.** The 30-year cumulative risk estimates (%) of CRC for 50-year-old women, according to the use of estrogen-only and quartiles of PRS. CRC, colorectal cancer; PRS.Q, the quartiles of polygenic risk score; MHT, menopausal hormone therapy; E-only, estrogen-only therapy

**
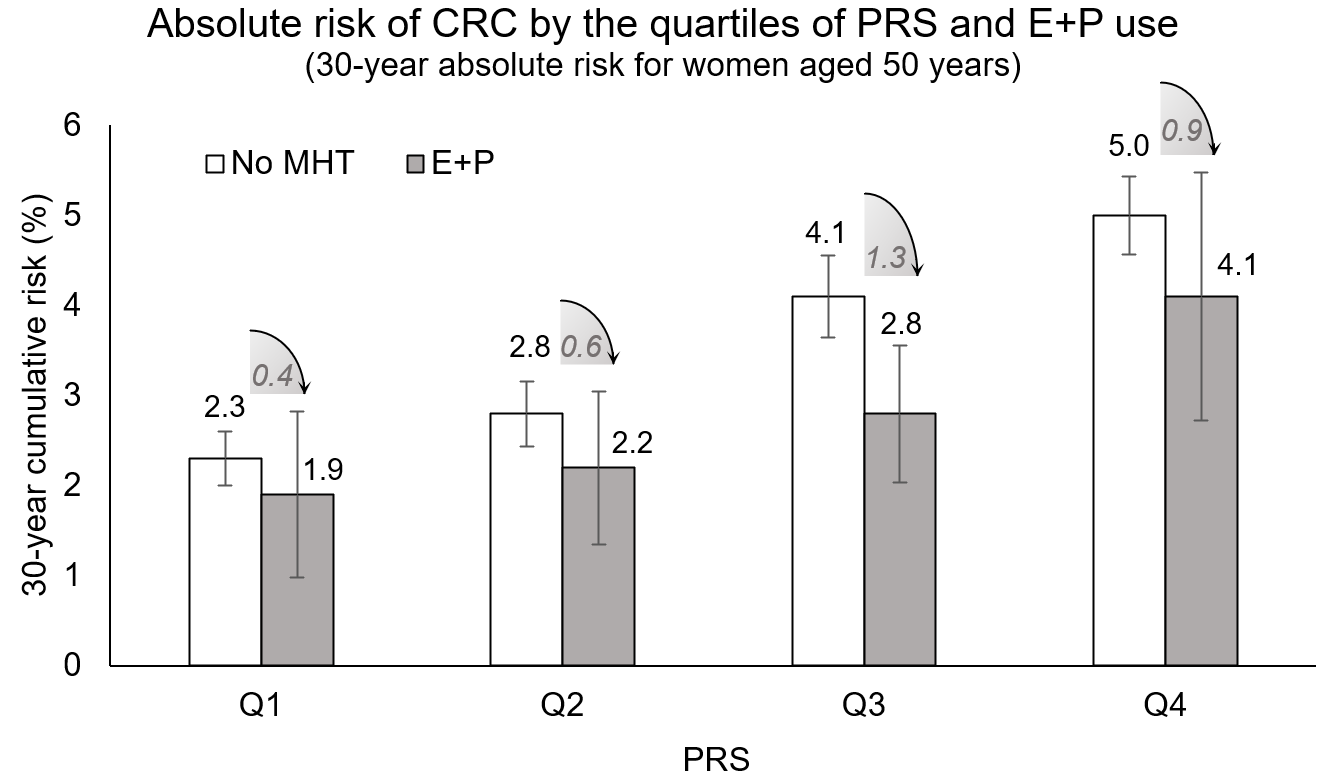
**

**Supplementary** **Figure 7.** The 30-year cumulative risk estimates (%) of CRC for 50-year-old women, according to the use of combined estrogen-progestogen therapy and quartiles of PRS. CRC, colorectal cancer; PRS.Q, the quartiles of polygenic risk score; MHT, menopausal hormone therapy; E+P, combined estrogen-progestogen therapy
