# Supplementary material for: Genetic risk impacts the association of menopausal hormone therapy with colorectal cancer risk
Source: Br J Cancer. 2024 Apr 1;130(10):1687–96. doi: 10.1038/s41416-024-02638-2 (PMC11091089; doi:10.1038/s41416-024-02638-2)
Supplement: Supplementary file 2 — Study description [file 41416_2024_2638_MOESM2_ESM.docx]

**Summary descriptions and study participant inclusion/exclusion criteria for each of studies included in our study “Genetic risk impacts the association of menopausal hormone therapy with colorectal cancer risk”**

**Study description**

Colon Cancer Family Registry (CCFR)

Campaign Against Cancer and Heart Disease (CLUE II)

Hawaii Colorectal Cancer Studies 2 & 3 (Colo2&3)

Cancer Prevention Study II (CPS II)

Colorectal Cancer Genetics & Genomics (CRCGEN)

Darmkrebs: Chancen der Verhütung durch Screening (DACHS)

Diet, Activity, and Lifestyle Study (DALS)

European Prospective Investigation into Cancer (EPIC)

Epidemiologische Studie zu Chancen der Verhütung, Früherkennung und optimierten THerapie chronischer ERkrankungen in der älteren Bevölkerung (ESTHER_VERDI)

Kentucky Case-Control Study (Kentucky)

Leeds Colorectal Cancer Study (LCCS)

Melbourne Collaborative Cohort Study (MCCS)

Multiethnic Cohort Study (MEC)

Molecular Epidemiology of Colorectal Cancer Study (MECC)

The North Carolina Colon Cancer Studies (NCCCS I/II)

Newfoundland Case-Control Study (NFCCR)

Nurses’ Health Study (NHS)

Nurses’ Health Study II (NHSII)

Prostate, Lung, Colorectal and Ovarian Cancer Screening Trail (PLCO)

Colon Cancer Pathways: Hyperplastic Polyps and Adenomas (REACH)

Swedish Mammography Cohort (SMC) and Swedish Men Cohort (SMC_COSM)

UK Biobank (UKB)

University of Southern California Hormone Replacement Therapy Colorectal Cancer Study (USC-HRT-CRC)

Cancer Screening Trial VITamins And Lifestyle cohort (VITAL)

The Women's Health Initiative (WHI)

**Colon Cancer Family Registry (CCFR)**

CCFR is a National Cancer Institute–supported consortium consisting of 6 centers dedicated to the establishment of a comprehensive collaborative infrastructure for interdisciplinary studies in the genetic epidemiology of colorectal cancer. The CCFR includes data from approximately 42,500 total subjects (10,500 probands and 26,800 unaffected and affected relatives,4,276 unrelated population-based controls and 923 spouse controls). Colorectal cancer (CRC) cases and controls, age 20 –74 years, were recruited at the 6 participating centers beginning in 1998. All participants completed a standardized questionnaire that asked about established and suspected risk factors for colorectal cancer, which included questions on medical history and medication use, reproductive history (for female participants), family history, physical activity, demographics, alcohol and tobacco use, and dietary factors. The **CCFR set 1 scan** (Illumina Human 1M or Human 1M-Duo) included population-based cases and unrelated population-based controls from the 3 population-based centers: Seattle Familial Colon Cancer Family Registry (SFCCR) at Fred Hutchinson Cancer Research Center, Ontario Familial Colorectal Cancer Registry (OFCCR) at Mount Sinai Hospital (previously at Cancer Care Ontario), and the Australasian Colorectal Cancer Family Registry (ACCFR) at the University of Melbourne). Cases were genetically enriched by oversampling those with a young age at onset or a positive family history of CRC. Controls were matched to cases on age and sex. The Set 2 scan includes population-based cases and matched controls from all six Colon CFR centers including Mayo Clinic, Rochester, Minnesota; the University of Hawaii, Honolulu, Hawaii; University of Southern California consortium, Los Angeles, California; Fred Hutchinson Cancer Research Center, Seattle, Washington; Mount Sinai Hospital, Toronto, Ontario; and The University of Melbourne, Victoria, Australia. As with Set 1, cases were genetically enriched by over-sampling those with a young age at onset or positive family history. Controls were same generation family controls. The **CCFR set 3 scan** (Affymetrix Axiom CORECT Set array) included CRC-affected population based probands and clinic-based cases and matched controls from 5 Colon CFR centers (excluding HCCFR). Controls were related family controls or unrelated population-based controls. All participants selected for CCFR sets-1, -2 and -3 were non-Hispanic White or of European ancestral heritage, which was confirmed with genotype data. In our study, however, **CCFR set 2** was removed from GxE subset because it’s a family-based study.

**Campaign Against Cancer and Heart Disease (CLUE II)**

The Campaign Against Cancer and Heart Disease, is a prospective cohort designed to identify biomarkers and other factors associated with risk of cancer, heart disease, and other conditions. 32,894 participants were recruited from May through October 1989 from Washington County, Maryland and surrounding communities. Colorectal cancer cases (n = 297) and matched controls (n = 296) were identified between 1989 and 2000 among participants in the CLUE II cohort of Washington County, Maryland.

**Hawaii Colorectal Cancer Studies 2 & 3 (Colo2&3)**

Patients with colorectal cancer were identified through the rapid reporting system of the Hawaii SEER registry and consisted of all Japanese, Caucasian, and Native Hawaiian residents of Oahu who were newly diagnosed with an adenocarcinoma of the colon or rectum between January 1994 and August 1998. Control subjects were selected from participants in an on-going population-based health survey conducted by the Hawaii State Department of Health and from Health Care Financing Administration participants. Controls were matched to cases by sex, ethnicity, and age (within two years). Personal interviews were obtained from 768 matched pairs, resulting in a participation rate of 58.2% for cases and 53.2% for controls. A questionnaire, administered during an in-person interview, included questions about demographics, lifetime history of tobacco, alcohol use, aspirin use, physical activity, personal medical history, family history of colorectal cancer, height and weight, diet (FFQ), and postmenopausal hormone use. A blood sample was obtained from 548 (71%) of interviewed cases and 662 (86%) of interviewed controls. SEER staging information was extracted from the Hawaii Tumor Registry. self-reported Caucasian subjects with DNA, and clinical and epidemiologic data were selected for genotyping.

**Cancer Prevention Study II (CPS II)**

The CPS II Nutrition cohort is a prospective study of cancer incidence and mortality in the United States, established in 1992 and described in detail elsewhere (Calle et al., 2002) [PMID: 12015775]. At enrollment, participants completed a mailed self-administered questionnaire including information on demographic, medical, diet, and lifestyle factors. Follow-up questionnaires to update exposure information and to ascertain newly diagnosed cancers were sent biennially starting in 1997. Reported cancers were verified through medical records, state cancer registry linkage, or death certificates. The Emory University Institutional Review Board approves all aspects of the CPS II Nutrition Cohort. A total of 360 cases and 359 controls were selected for this study.

**Colorectal Cancer Genetics & Genomics (CRCGEN)**

The Spanish study combines data of three case-control studies. The first one, performed in University Hospital of Bellvitge, L'Hospitalet, Barcelona, recruited 304 incident pathology- confirmed CRC cases and 293 age and sex frequency-matched hospital controls during the period 1996-1998. The control group consisted of patients without previous colorectal cancer who had been randomly selected among those admitted to the same hospital during the same period. To avoid selection bias, the criterion of inclusion in the control group was a new diagnosis. The second study, performed in the same hospital during the period 2007-2015, included a total of 324 cases and 376 population controls. The control group consisted of subjects invited to participate and selected from the primary health care lists of the hospital’s referral area, frequency matched by age and sex. The third study was conducted in Hospital of Leon, Leon, during 2008-2013. A total of 325 incident CRC cases and 407 population controls were included. The control population consisted of subjects invited to participate and selected from the primary health care lists, frequency matched by age and sex. Written informed consent was required from all participants. Each hospital's ethics committees (Bellvitge and Leon) approved the protocols of the study.

**Darmkrebs: Chancen der Verhütung durch Screening (DACHS)**

This German study was initiated as a large population-based case-control study in 2003 in the Rhine-Neckar-Odenwald region (southwest region of Germany) to assess the potential of endoscopic screening for reduction of CRC risk and to investigate etiologic determinants of disease, particularly lifestyle/environmental factors and genetic factors. During an in-person interview, data were collected on demographics, medical history, family history of CRC, and various life-style factors, as were blood and mouthwash samples. Cases with a first diagnosis of invasive CRC (International Classification of Diseases 10 codes C18-C20) who were at least 30 years of age (no upper age limit), German speaking, a resident in the study region, and mentally and physically able to participate in a one-hour interview, were recruited by their treating physicians either in the hospital a few days after surgery, or by mail after discharge from the hospital. Cases were confirmed based on histologic reports and hospital discharge letters following diagnosis of CRC. All hospitals treating CRC cancer patients in the study region participated. Community-based controls were randomly selected from population registries, employing frequency matching with respect to age (5-year groups), sex, and county of residence. Controls with a history of CRC were excluded. Controls were contacted by mail and follow-up calls. During an in-person interview, data were collected on demographics, medical history, family history of CRC, and various lifestyle factors, as were blood and mouthwash samples. The set 1 scan consisted of a subset of participants recruited up until 2007, and samples were frequency-matched on age and sex. The set 2 scan consisted of additional subjects who were recruited up until 2010 as part of this ongoing study. The set 3 consist of 1,210 cases and 617 matched controls genotyped using the HumanOmniExpressExome-8v1-2 array, and set 4, an additional 1,013 cases and 657 matched controls, were genotyped using the Infinium OncoArray-500K.

**Diet, Activity, and Lifestyle Study (DALS)**

DALS was a population-based, case–control study of colon cancer.3 Participants were recruited between 1991 and 1994 from 3 locations: the Kaiser Permanente Medical Care Program of Northern California, an 8-county area in Utah, and the metropolitan Twin Cities area of Minnesota. Eligibility criteria for cases included age at diagnosis between 30 and 79 years, diagnosis with first primary colon cancer (International Classification of Disease for Oncology, Second Edition, 18.0 and 18.2–18.9) between October 1, 1991, and September 30, 1994, English speaking, and competency to complete the interview. Individuals with cancer of the rectosigmoid junction or rectum were excluded, as were those with a pathology report noting familial adenomatous polyposis, Crohn’s disease, or ulcerative colitis. A rapid-reporting system was used to identify all incident cases of colon cancer, resulting in the majority of cases being interviewed within 4 months of diagnosis. Controls from the Kaiser Permanente Medical Care Program were selected randomly from membership lists. In Utah, controls younger than 65 years of age were selected randomly through random-digit dialing and driver license lists. Controls 65 years of age and older were selected randomly from Health Care Financing Administration lists. In Minnesota, controls were identified from Minnesota driver license or state identification lists. Cases and controls were matched by 5-year age groups and sex. The set I scan consisted of a subset of the study, from Utah, Minnesota, and the Kaiser Permanente Medical Care Program, and was restricted to subjects who self-reported as white non-Hispanic. The set 2 scan consisted of subjects from Utah and Minnesota who were not genotyped in set 1. Set 2 was restricted to subjects who self-reported as white non-Hispanic and those who had appropriate consent to post data to the database of Genotypes and Phenotypes.

**European Prospective Investigation into Cancer (EPIC)**

EPIC is an on-going multicenter prospective cohort study designed to investigate the associations between diet, lifestyle, genetic and environmental factors and various types of cancer. In summary, 521,448 participants (~70% women) mostly aged 35 years or above were recruited between 1992 and 2000. Participants were recruited from 23 study centers in ten European countries. The current study included participants from France, Germany, Greece, Italy, the Netherlands, Spain, Sweden, and United Kingdom (UK). Blood samples were collected at baseline according to standardized procedures, and stored at the International Agency for Research on Cancer (IARC; -196°C, liquid nitrogen) for all countries except Sweden (-80°C freezers). All study participants provided written informed consent. Ethical approval for the EPIC study was obtained from the review boards of IARC and local participating centers. Incident cancer cases were identified using population cancer registries in Italy, the Netherlands, Spain, and the United Kingdom. In Sweden (only the Umeå site was included), cases were identified by linkage with the essentially complete Cancer Registry of Northern Sweden and were verified by a gastrointestinal pathologist. In France, Germany and Greece, cancer cases were identified during follow-up by a combination of methods including: health insurance records, cancer and pathology registries, and by active follow-up directly through study participants or through next-of-kin. Controls were selected from the full cohort of individuals who were alive and free of cancer (except non-melanoma skin cancer) at the time of diagnoses of the cases, using incidence density sampling and matched by: age (±6 months at recruitment), sex, study center, follow-up time since blood collection, time of day at blood collection (±4 hours), fasting status, menopausal status, and phase of menstrual cycle at blood collection. In total, 2,095 incident colorectal cancer cases, and 2,306 matched controls were genotyped using the HumanOmniExpressExome-8v1-2 array.

**Epidemiologische Studie zu Chancen der Verhütung, Früherkennung und optimierten THerapie chronischer ERkrankungen in der älteren Bevölkerung (ESTHER_VERDI)**

In the ESTHER/VERDI study, patients diagnosed with various forms of cancer at ages 50-75, including patients with colorectal cancer (n=420), were recruited statewide in Saarland, Germany between 1996-1998 and 2001-2003. Controls, who were frequency matched by sex and age, were randomly drawn from women and men who were recruited for a statewide cohort study in Saarland, Germany when undergoing a health check-up with their general practitioners in 2000-2002 (n=437). Blood samples were drawn by the treating physicians who also provided medical data from their records. Risk factor information was collected by self-administered standardized questionnaires. The analytic dataset from the ESTHER/VERDI study included in the Discovery GWAS consisted of 420 CRC cases and 437 controls.

**Kentucky Case-Control Study (Kentucky)**

Control study was initiated in July 2003 through the University of Kentucky Cancer Center. A web-based reporting system implemented by the Kentucky Cancer Registry in 2003 has facilitated rapid report of cases state- wide, with approximately 76.8% of all cases reported to the registry within 6 months of diagnosis. Cases (>21 years) diagnosed with histologically confirmed colon cancer and entered into the registry within 6 months of their diagnoses are invited to join the study. Population-based unrelated controls are recruited through random digit dialing and are frequency matched to the cases by age (±5 years), gender, and race. Excluded from the study are those individuals who have been diagnosed with colon cancer because of known hereditary forms of colon cancer or polyposis such as familial adenomatous polyposis (FAP), hereditary non-polyposis colorectal cancer (HNPCC), Peutz-Jeghers, and Cowden disease. Currently there are more than 1,040 incident population-based cases of colorectal cancer and 1,750 population-based controls fully recruited, with comprehensive epidemiologic data, pathology data, and DNA from cases and controls.

**Leeds Colorectal Cancer Study (LCCS)**

Following local ethical approval, colorectal cancer cases were recruited from 1997 until 2012 in Leeds, UK through surgical clinics. Initially funding was provided by the UK Ministry of Agriculture, Farming and Fisheries (subsequently the Food Standards Agency) and Imperial Cancer Research Fund (subsequently Cancer Research UK). Recruitment also occurred similarly in Dundee, Perth and York between the periods of 1997 and 2001 using the same protocol and the data and samples were combined. Pathologically confirmed cases were consented at outpatient clinics, providing information on known and postulated risk factors for colorectal cancer (diet, lifestyle and family history) as well as providing a blood sample for DNA. Exclusion criteria included pre-existing diverticular disease and an inability to complete the questionnaire. The General Practitioners of cases (all UK residents have a nominated General Practitioner to whom to refer initial medical queries) and these GPs were asked to send letters to other persons on their patient list of the same gender and born within 5 years of the case. Subsequently to enhance the number of controls, we systematically invited patients from selected GP practices. Diet was assessed in cases and controls using an extensive dietary and lifestyle questionnaire modified by that produced by the European Prospective Investigation in Cancer (EPIC). The frequency that each specific food items were eaten was recorded and we also obtained average fruit and vegetable consumption as a cross-check. In total, 1591 cases and 739 controls provided a DNA sample.

**Melbourne Collaborative Cohort Study (MCCS)**

The MCCS is a prospective study that recruited 41,514 healthy adult volunteers (17,045 men) aged between 27 and 76 years (99% aged 40-69) from the Melbourne metropolitan area between 1990 and 1994. All CRC cases eligible for this study were selected based on the availability of a blood sample, were not genotyped previously, had no pre-baseline history of Victorian Cancer Registry (VCR) confirmed CRC or pre-baseline history of another primary cancer, excluding non-melanocytic skin cancer. Incident cases of invasive (including metastatic) adenocarcinoma of the colon or rectum were identified through the VCR up to 31^st^ December 2012. Germline DNA was extracted from blood samples. Study participants provided written, informed consent in accordance with the Declaration of Helsinki. The study was approved by Cancer Council Victoria’s Human Research Ethics Committee and performed in accordance with the institution’s ethical guidelines. Set 1 consisted of 576 incident cases diagnosed during follow-up from baseline (1990-1994) till mid-2010 and 576 individually matched population-based controls22. The matching factors were sex, country of birth (Australia/UK, Italy and Greece), and year of baseline attendance. Cases were all incident cases in the cohort ascertained through linkage to the Victorian Cancer Registry and other State cancer registries in Australia. For the GWAS, cases with only DNA extracted from Guthrie cards available were excluded. Samples were genotyped on the Affymetrix Axiom CORECT Set array. Set 2 consisted of 238 CRC cases met our eligibility criteria and were matched to a control using risk set sampling with age as the time variable. Controls were matched to cases based on sex, year of baseline attendance and country of birth (Australia/New Zealand/United Kingdom/Greece/Italy/other). Samples were genotyped on OncoArray.

**Multiethnic Cohort Study (MEC)**

MEC was initiated in 1993 to investigate the impact of dietary and environmental factors on major chronic diseases, particularly cancer, in ethnically diverse populations in Hawai’i and California. The study recruited 96,810 men and 118,441 women aged 45 to 75 years between 1993 and 1996. Incident colorectal cancer cases occurring since January 1995, and controls were contacted for blood or saliva samples. The median interval between diagnosis and blood draw was 14 months (interquartile range, 10-19) among cases and the participation rate 74%. A sample of cohort participants was randomly selected to serve as controls at the onset of the nested case-control study (participation rate 66%). The selection was stratified by sex, age, and race/ethnicity. Colorectal cancer cases are identified through the Rapid Reporting System of the Hawai’i Tumor Registry and through quarterly linkage to the Los Angeles County Cancer Surveillance Program. Both registries are members of SEER. Set 1, in GECCO, self-reported White subjects from the nested case-control study described above with DNA, and clinical and epidemiologic data were selected for genotyping. Set 2 were genotyped on OncoArrary.

**Molecular Epidemiology of Colorectal Cancer Study (MECC)**

The Molecular Epidemiology of Colorectal Cancer Study (MECC) is a population-based case-control study of colorectal cancer (CRC). Incident, pathologically-confirmed CRC cases and controls were recruited from a specific region of northern Israel. Participant recruitment began in 1998 and remains on-going. Individually-matched controls with no prior history of CRC are selected from the same source population that gave rise to cases using the Clalit Health Services database. Matching factors include age, sex, Jewish ethnicity (Jew versus non-Jew), and primary clinic site. Subjects are interviewed for demographic and clinical information, family history, and dietary habits, gave a venous blood sample, and provided permission for tumor tissue retrieval. Written, informed consent was obtained according to Institutional Review Board-approved protocols at Carmel Medical Center in Haifa and the University of Southern California (HS-12-00324, HS-12-00672, and HS-08-00378). Germline DNA was extracted from whole blood for genotyping. Set 1, a case-control set consisted of 484 cases and 498 controls genotyped on the Illumina Omni 2.5 array. Case selection for genotyping in MECC1 enriched for colon cancer, enriched for a specific stage distribution for a separate GWAS study of stage and prognosis, and excluded cases with microsatellite instable (MSI-H) tumors. Set 2 utilizes genotypes from 1,120 cases and 1156 controls on the Affymetrix Axiom CORECT Set array. Cases were unselected for cancer site, stage, or MSI. In addition to self-reported ancestral heritage (Ashkenazi / Sephardi), PCA analysis was used to examine the correspondence between self-reported ancestry and genotypic classification. Set 3 consisted of 3,591 cases of pathologically-confirmed adenocarcinoma and 2,848 controls on the OncoArray.

**The North Carolina Colon Cancer Studies (NCCCS I/II)**

The North Carolina Colon Cancer Studies (NCCCS I- colon and NCCCS II-rectal) were population-based case-control studies conducted in 33 counties of North Carolina. Cases were identified using the rapid case ascertainment system of the North Carolina Central Cancer Registry. Patients with a first diagnosis of histologically confirmed invasive adenocarcinoma of the colon (cecum through sigmoid colon) between October 1996 and September 2000 were classified as potential cases in the NCCCS I. The NCCCS II included patients with a first diagnosis of histologically confirmed invasive adenocarcinoma of the sigmoid colon, rectosigmoid, or rectum (hereafter collectively referred to as rectal cancer) between May 2001 and September 2006. Additional eligibility requirements were: aged 40–80 years, residence in one of the 33 counties, ability to give informed consent and complete an interview, had a driver’s license or identification card issued by the North Carolina Department of Motor Vehicles (if under the age of 65), and had no objections from the primary physician in regard to contacting the individual. Controls, identified and sampled during the respective study dates, were selected from two sources. Potential controls under the age of 65 were identified using the North Carolina Department of Motor Vehicles records. For those 65 years and older, records from the Center for Medicare and Medicaid Services were used. Controls were matched to cases using randomized recruitment strategies. Recruitment probabilities were done using strata of 5-year age, sex, and race groups. Dietary information was collected using a modified version of the semiquantitative food frequency questionnaire developed at the National Cancer Institute. In addition, participants were asked about vitamin and mineral supplementation, special diets, restaurant eating, sodium use, and fats used in cooking. In NCCCS I, 515 colorectal cases and 687 matched controls were sent for genotyping. In NCCCS II, 796 colorectal cases and 823 controls were sent from the NCCCS II for genotyping. Controls were matched to CRC cases as 1:1 ratio. Matching was done on age, race, and sex. Age was matched on ±5 years. Race and sex was matched exactly. For the cases without matched controls, matching was done only on sex and race.

**Newfoundland Case-Control Study (NFCCR)**

The NFCCR is a case-control study that includes pathology confirmed CRC cases less than 75 years of age diagnosed between January 1999 and December 2003, as identified from the Newfoundland Cancer Registry. The Newfoundland Cancer Registry registers all cases of invasive cancer diagnosed among residents of the province of Newfoundland and Labrador. Consenting patients received a family history questionnaire and were asked to provide a blood sample and to permit access to tumor tissue and medical records. If a patient was deceased, we sought the participation of a close relative for the purposes of obtaining the family history and for permission to access tissue blocks and medical records. Use of proxies in this way removes the bias of excluding advanced stage patients who die before they can give consent. Population- based controls were identified by random digit dialing from the residents of the province and matched to the cases on sex and five-year age groups. Controls provided a blood sample and filled out a risk factor questionnaire. Set 1, cases only genotyped on Illumina OmniQuad. Set 2 were genotyped on the Affymetrix Axiom CORECT Set array.

**Nurses’ Health Study (NHS)**

The NHS cohort began in 1976 when 121,700 married female registered nurses age 30–55 years returned the initial questionnaire that ascertained a variety of important health-related exposures.1 Since 1976, follow-up questionnaires have been mailed every 2 years. Colorectal cancer and other outcomes were reported by participants or next-of-kin and followed up through review of the medical and pathology record by physicians. Overall, more than 97% of self-reported colorectal cancers were confirmed by medical-record review. Information was abstracted on histology and primary location. The rate of follow-up evaluation has been high: as a proportion of the total possible follow-up time, follow-up evaluation has been more than 92%. Colorectal cancer cases were ascertained through June 1, 2008. In 1989 –1990, 32,826 women in NHS I mailed blood samples by overnight courier, which were aliquoted into buffy coat and stored in liquid nitrogen. In 2001–2004, 29,684 women in NHS I who did not previously provide a blood sample mailed a swish-and-spit sample of buccal cells. Incident cases were defined as those occurring after the subject provided a blood or buccal sample. Prevalent cases were defined as those occurring after enrollment in the study in 1976 but before the subject provided either a blood or buccal sample. After excluding participants with histories of cancer (except nonmelanoma skin cancer), ulcerative colitis, or familial polyposis, 2 case-control sets were constructed from which DNA was isolated from either buffy coat or buccal cells for genotyping: (1) set 1, a case-control set with cases of colorectal cancer matched to randomly selected controls who provided a blood sample and were free of colorectal cancer at the same time the colorectal cancer was diagnosed in the case; and (2) set 2, a case-control set with cases of colorectal cancer matched to randomly selected controls who provided a buccal sample and were free of colorectal cancer at the same time the colorectal cancer was diagnosed in the cases. For both case-control sets, matching criteria included year of birth (within 1 year) and month/year of blood or buccal cell sampling (within 6 months). Cases were pair matched 1:1, 1:2, or 1:3 with a control participant(s). In addition to colorectal cancer cases and controls, a set of adenoma cases and matched controls with available DNA from buffy coat were selected for genotyping. Over the follow-up period, data were collected on endoscopic screening practices and, if individuals had been diagnosed with a polyp, the polyps were confirmed to be adenomatous by medical record review. Adenoma cases were 1ascertained through June 1, 2008. A separate case-control set (set 3) was constructed of participants diagnosed with advanced adenoma matched to control participants who underwent a lower endoscopy in the same time period and did not have an adenoma. Advanced adenoma was defined as an adenoma more than 1 cm in diameter and/or with tubulovillous, villous, or high-grade dysplasia/carcinoma-in-situ histology. Matching criteria included year of birth (within 1 year) and month/year of blood sampling (within 6 months), the reason for their lower endoscopy (screening, family history, or symptoms), and the time period of any prior endoscopy (within 2 years). Controls matched to cases with a distal adenoma either had a negative sigmoidoscopy or colonoscopy examination, and controls matched to cases with proximal adenoma all had a negative colonoscopy. Set 4, colorectal cancer cases were ascertained through June 1, 2012 and excluded cases included in the previous sets. Participants with histories of cancer (except nonmelanoma skin cancer), ulcerative colitis, or familial polyposis were excluded. CRC cases matched to randomly selected controls who provided a blood or buccal sample and were free of colorectal cancer at the same time the colorectal cancer was diagnosed in the cases. Matching criteria included year of birth (within 1 year) and month/year of blood or buccal cell sampling (within 1 year). If no control could be matched for a case using the initial stringent criteria, age criteria were relaxed to <5 years to find an eligible control. A total of 315 CRC cases and 313 controls were included and all participants were genotyped using the HumanOmniExpressExome-8v1-2 array. Set 5, a separate case-control set was constructed of participants diagnosed with advanced adenoma matched to control participants who underwent a lower endoscopy in the same time period and did not have an adenoma. Advanced adenoma was defined as an adenoma 1 cm or larger in diameter and/or with tubulovillous, villous, or high-grade dysplasia/carcinoma-in-situ histology. Matching criteria included year of birth (within 1 year) and month/ year of blood sampling (within 6 months), the reason for their lower endoscopy (screening, family history, or symptoms), and the time period of any prior endoscopy (within 2 years). Controls matched to cases with a distal adenoma either had a negative sigmoidoscopy or colonoscopy examination, and controls matched to cases with proximal adenoma all had a negative colonoscopy. In total, 254 advanced adenoma cases and 225 controls were selected for genotyping.

**Nurses’ Health Study II (NHSII)**

The Nurses' Health Study II (NHSII) is an ongoing cohort of 116,430 female registered nurses in the US, aged 25-42 years at baseline in 1989. Demographic, lifestyle and health-related information were obtained from participants at baseline and updated every 2 years using self-administered questionnaires. The follow-up rate in each cycle has been over 90% to date. Study participants who had not previously reported a diagnosis of cancer and had responded to the 1995 NHSII study questionnaire were invited to provide blood samples between 1996 and 1999. Blood samples were collected from 29,611 NHSII participants, aged 32 to 54 years at the time of blood draw. Similarly, between 2004 and 2006, active study participants who had not previously provided a blood sample were invited to provide buccal samples. Swish-and-spit sample of buccal cells were received from 29,859 participants. Cases and controls selected for genotyping were nested within the subcohort of participants who provided a blood or a buccal sample. Participants with a prior history of any cancer (except non-melanoma skin cancer), ulcerative colitis, or familial polyposis syndromes were excluded. Incident cases of colorectal adenocarcinoma were ascertained first by self-report and later confirmed by reviewing medical records and pathology reports within each follow up cycle. Deaths due to colorectal cancer were identified through family or next of kin or by querying the National Death Index. Controls were randomly selected among participants in the subcohort provided they were free of colorectal cancer and matched to a corresponding case by both age (within 1 year) and sample collection date (month/year of blood or buccal sampling). Overall, 133 cases and 132 matched controls were selected for OncoArray genotyping, and 109 cases and 102 controls with ≥80% estimated European ancestry based on STRUCTURE were included in the Discovery GWAS.

**Prostate, Lung, Colorectal and Ovarian Cancer Screening Trail (PLCO)**

PLCO enrolled 154,934 participants (men and women, aged between 55 and 74 years) at ten centers into a large, randomized, two-arm trial to determine the effectiveness of screening to reduce cancer mortality. Sequential blood samples were collected from participants assigned to the screening arm. Participation was 93% at the baseline blood draw. In the observational (control) arm, buccal cells were collected via mail using the "swish-and-spit" protocol and participation rate was 65%. Details of this study have been previously described and are available online (http://dcp.cancer.gov/plco). The set 1 scan included a subset of 577 colon cancer cases self-reported as being non-Hispanic white with available DNA samples, questionnaire data, and appropriate consent for ancillary epidemiologic studies. Cases were excluded if they had a history of inflammatory bowel disease, polyps, polyposis syndrome, or cancer (excluding basal or squamous cell skin cancer). Controls originated from the Cancer Genetic Markers of Susceptibility prostate cancer scan18,19 (all male) and the GWAS of Lung Cancer and Smoking20 (enriched for smokers), along with an additional 92 non-Hispanic white female controls. For the set 2 scan, cases were individuals with colorectal cancer from both arms of the trial who were not already included in set 1. Samples were excluded if participants did not sign appropriate consent forms, if DNA was unavailable, if baseline questionnaire data with follow-up evaluation were unavailable, if they had a history of colon cancer before the trial, if they had a rare cancer, if they were already in a colon GWAS, or if they were a control in the prostate or lung populations. Controls were frequency-matched 1:1 to cases without replacement, and cases were not eligible to be controls. Matching criteria were age at enrollment (2-year blocks), enrollment date (2-year blocks), sex, race/ethnicity, trial arm, and study year of diagnosis (i.e., controls must be cancer free into the case’s year of diagnosis).

**Colon Cancer Pathways: Hyperplastic Polyps and Adenomas (REACH)**

A case-control design was conducted within participants enrolled in the Group Health Cooperative, an integrated health-care provider in Washington State, aged 20–79 years who underwent an index colonoscopy for any indication between 1998 and 2007 and were diagnosed on the basis of clinical pathology with adenomas (Classification of Diseases, Ninth Revision (ICD-9), code 211.3) and/or hyperplastic polyps (ICD-9 code 211.4) or who were polyp-free (controls). Eligible persons had been enrolled in the Group Health Cooperative for at least 3 years and had not undergone a prior colonoscopy within 1 year of the index colonoscopy. Participants with poor bowel preparation at the index colonoscopy and those with a prior or new diagnosis of colorectal cancer, familial colorectal cancer syndromes (such as familial adenomatous polyposis), or other colorectal disease were ineligible. A systematic sample of eligible colonoscopy patients was recruited, and approximately 75% agreed to participate and provided written informed consent. Participants completed a structured and polyps were evaluated via standardized pathology review. Study protocols were approved by the institutional review boards of the Group Health Cooperative and the Fred Hutchinson Cancer Research Center (Seattle, Washington).

**Swedish Mammography Cohort (SMC) and Swedish Men Cohort (SMC_COSM)**

The Swedish Mammography Cohort (SMC) and the Cohort of Swedish Men (COSM) are two large population-based prospective cohorts from central Sweden. The SMC was initiated between 1987 and 1990 when all women born in 1914-1948 and residing in Uppsala and Västmanland counties were invited; response rate 74% (n=66,651). The COSM started in late 1997, with the invitation of all men born in 1918-1952 and residing in Västmanland and Örebro county; response rate 49% (n=48,850). Questionnaire data on diet and other lifestyle factors was collected at the start of the studies and has been updated repeatedly during follow-up. Further, biological samples (saliva, blood) have been collected together with signed informed consent and are available for DNA extraction. The cohorts are annually matched to the Swedish Cancer Register for ascertainment of incident cancer cases. For the CORECT study, follow-up through 2011 was available. The Regional Ethical Review Board at Karolinska Institutet in Stockholm approved genetic studies of CRC based on the cohorts. The analytic dataset from this study included in the Discovery GWAS consisted of 580 CRC cases and 859 controls.

**UK Biobank (UKB)**

We constructed a CRC and advanced adenoma nested case-control dataset from the UK Biobank resource (application number 8614). CRC cases were defined as subjects with primary invasive CRC diagnosed, or who died from CRC according to ICD9 (1530-1534, 1536-1541) or ICD10 (C180, C182-C189, C19, C20) codes. Appendix cases, non-invasive (in situ) CRC cases, cases with histology of tumor as carcinoid, and related tumors and lymphomas (ICD-O-3 tumor histology codes 8240-8249, 9590-9729) were excluded. Advanced adenoma cases were defined as primary in situ CRC cases according to ICD9 (2303, 2304) or ICD10 (D010-D012) codes, or benign neoplasms according to ICD10 codes (D120, D122, D123, D124-D128, D374, D375) with ICD-O-3 tumor histology codes 8210, 8211, 8220, 8221, or 8261-8263. Incident and prevalent CRC or advanced adenoma cases were defined based on date of diagnosis and date of enrollment. Eligible control participants were required to be free of invasive colorectal cancer, non-invasive (in situ) CRC, appendix, anus, anal canal, and overlapping lesion of rectum, anus and anal canal cancer, or advanced adenoma. For incident cases, each case was matched with 4 controls that exactly matched the following matching criteria: age at enrollment, year at enrollment, race/ethnicity, and sex. Control selection was done in a time-forward manner, selecting one control for each case, first from the risk set at the time of the case’s event, and then multiple passes were made to match second, third and fourth controls. For prevalent cases, each case was matched with 4 controls that exactly matched the following matching criteria: year at enrollment, race/ethnicity, and sex. The risk set was then defined as controls who were at risk at the age when the cases were diagnosed. For matching of both incident and prevalent cases, the matching algorithm selected the closest match based on criteria to minimize an overall distance measure [50]. In total, 5,356 CRC (5,004) or advanced adenoma (352) cases and 21,407 matched controls were included in the replication analysis. All participants were genotyped using the Affymetrix UK Biobank Axiom Array.

**University of Southern California Hormone Replacement Therapy Colorectal Cancer Study (USC-HRT-CRC)**

Observational epidemiological studies and randomized trials have reported a protective effect of estrogen and progestin therapy (EPT) on the risk of colorectal cancer, but the findings on estrogen-alone therapy (ET) are less consistent. To further investigate the relationship between menopausal hormones and risk of colon cancer, we conducted a population-based case–control study in Los Angeles County involving 831 women with newly diagnosed colon cancer and 755 population-based control women. The cases were identified by the Los Angeles County Cancer Surveillance Program, part of the National Cancer Institute’s Surveillance, Epidemiology and End Results Program. Eligible subjects were English-speaking women with a histologically confirmed primary colon cancer diagnosed between the ages of 55 and 74 years on or after January 1998 through December 2002 and who were residents of Los Angeles County. Race/ ethnicity and aged matched female controls were identified through a well-established neighborhood recruitment algorithm. In-person interviews were conducted using a structured questionnaire that covered medical, menstrual, and reproductive history, use of select hormonal and non-hormonal medications, body size, physical activity, and other lifestyle factors. Interviewed participants were asked to donate a blood specimen. DNA from buffy coats of peripheral blood samples were used for OncoArray genotyping. The analytic dataset from the USC-HRT-CRC study included in the Discovery GWAS consisted of 346 CRC cases and 409 controls.

**Cancer Screening Trial VITamins And Lifestyle cohort (VITAL)**

The VITamins And Lifestyle (VITAL) cohort comprises of 77,721 Washington State men and women aged 50 to 76 years, recruited from 2000 to 2002 to investigate the association of supplement use and lifestyle factors with cancer risk. Subjects were recruited by mail, from October 2000 to December 2002, using names purchased from a commercial mailing list. All subjects competed a 24-page questionnaire and buccal-cell specimens for DNA was self-collected by 70% of the participants. Subjects are followed for cancer by linkage to the western Washington SEER cancer registry and are censored when they move out of the area covered by the registry or at time of death. Details of this study have been previously described 22. In GECCO, a nested case-control set was genotyped. Samples included, colorectal cancer cases with DNA, excluding subject with colorectal cancer before baseline, in situ cases, (large cell) neuroendocrine carcinoma, squamous cell carcinoma, carcinoid tumor, Goblet cell carcinoid, any type of lymphoma, including non-Hodgkin, Mantle cell, large B-cell, or follicular lymphoma. Controls were matched on age at enrollment (within one year), enrollment date (within one year), sex, and race / ethnicity. One control was randomly selected per case among all controls that matched on the four factors above and where the control follow-up time was greater than follow-up time of the case until diagnosis.

**The Women's Health Initiative (WHI)**

The WHI (Women’sHealth Initiative) is a long-term health study of 161,808 post-menopausal women aged 50–79 years at 40 clinical centers throughout the United States. WHI comprised a clinical trial arm, an observational study (OS) arm, and several extension studies. The details of WHI have been described previously and are available online (https://www.whi.org). In GECCO, set 1 cases were selected from the September 12, 2005, database and comprised centrally adjudicated colon cancer cases from the OS arm who self-reported as white. Controls were first selected among controls previously genotyped as part of a hip fracture GWAS conducted within the WHI OS arm and matched to cases on age (within 3 years), enrollment date (within 365 days), hysterectomy status, and prevalent conditions at baseline. For 37 cases, there was no control match in the hip fracture GWAS. For these participants, we identified a matched control in the WHI OS arm based on the same criteria. In the set 2 scan, cases were selected from the August 2009 database and comprised centrally adjudicated colon and colorectal cancer cases from the OS and clinical trial arms who were not genotyped in set 1. In addition, case and control participants were subject to the following exclusion criteria: a prior history of colorectal cancer at baseline, institutional review board approval not available for data submission into dbGaP, and insufficient DNA available. Matching criteria included age (within years), race/ethnicity, WHI date (within 3 years), WHI Calcium and Vitamin D study date (within 3 years), and randomization arms (OS flag, hormone therapy assignments, dietary modification assignments, calcium/vitamin D assignments). In addition, they were matched by the 4 regions of randomization centers. Each case was matched with 1 control (1:1) who met the matching criteria exactly. Control selection was performed in a time-forward manner, selecting one control for each case first from the risk set at the time of the case’s event. The matching algorithm could select the closest match based on a criterion to minimize an overall distance measure. Each matching factor was given the same weight. Additional available controls who were genotyped as part of the hip fracture GWAS were included to improve power. In the set 3, cases were selected from the December 2015 database and comprised centrally adjudicated colon and colorectal cancer cases from the OS and clinical trial arms who were not genotyped in set 1 and 2. Control participants were required to be free of invasive colorectal cancer and non-invasive (stage 0 in situ) colorectal cancer. Controls can have missing history of colorectal cancer. Centrally denied cases of colorectal cancer were not allowed into the control pool. Case and control participants were subject to the following exclusion criteria: had prior history of colorectal cancer at baseline; cannot be deposited to dbGAP, except participants were in Stage 1 or hip fracture controls; lost to follow-up after enrollment (ie, participants didn’t have any follow-up visit after enrollment); unknown race/ethnicity.

**Study References**

**CCFR:**

Newcomb PA, Baron J, Cotterchio M*, et al.* Colon Cancer Family Registry: an international resource for studies of the genetic epidemiology of colon cancer. Cancer Epidemiol Biomarkers Prev 2007;16(11):2331-43.

Figueiredo JC, Lewinger JP, Song C*, et al.* Genotype-environment interactions in microsatellite stable/microsatellite instability-low colorectal cancer: results from a genome-wide association study. Cancer Epidemiol Biomarkers Prev 2011;20(5):758-66.

**CLUE II:**

Kakourou A, Koutsioumpa C, Lopez DS, Hoffman-Bolton J, Bradwin G, Rifai N, Helzlsouer KJ, Platz EA, and Tsilidis KK. Interleukin-6 and risk of colorectal cancer: results from the CLUE II cohort and a meta-analysis of prospective studies. *Cancer Causes Control* 2015;26:1449–1460.

**Colo2&3:**

Le Marchand L, Hankin JH, Wilkens LR, et al. Combined effects of well-done red meat, smoking, and rapid N-acetyltransferase 2 and CYP1A2 phenotypes in increasing colorectal cancer risk. Cancer Epidemiol Biomarkers Prev 2001;10:1259–1266.

**CPS II:**

Calle EE, Rodriguez C, Jacobs EJ, Almon ML, Chao A, McCullough ML, Feigelson HS, and Thun MJ. The American cancer society cancer prevention study II nutrition cohort—rationale, study design, and baseline characteristics. *Cancer* 2002;94:2490–2501.

Campbell PT, Deka A, Briggs P, Cicek M, Farris AB, Gaudet MM, Jacobs EJ, Newton CC, Patel AV, Teras LR, Thibodeau SN, Tillmans L, and Gapstur SM. Establishment of the Cancer Prevention Study II Nutrition Cohort Colorectal Tissue Repository. *Cancer Epidemiol Biomarkers Prev* 2014;23:2694-702.

**DACHS:**

Brenner H, Chang-Claude J, Seiler CM, et al. Protection from colorectal cancer after colonoscopy: population-based case-control study. Ann Intern Med 2011;154:22–30.

Lilla C, Verla-Tebit E, Risch A, et al. Effect of NAT1 and NAT2 genetic polymorphisms on colorectal cancer risk associated with exposure to tobacco smoke and meat consumption. Cancer Epidemiol Biomarkers Prev 2006;15:99–107.

**DALS:**

Slattery ML, Potter J, Caan B, et al. Energy balance and colon cancer–beyond physical activity. Cancer Res 1997;57:75–80.

**EPIC:**

Riboli, E. *et al.* European Prospective Investigation into Cancer and Nutrition (EPIC): study populations and data collection. *Public Health Nutr* 2002;5:1113–1124.

**ESTHER_VERDI:**

Jansen L, Herrmann A, Stegmaier C*, et al.* Health-related quality of life during the 10 years after diagnosis of colorectal cancer: a population-based study. J Clin Oncol 2011;29(24):3263-9.

Breitling LP, Raum E, Muller H*, et al.* Synergism between smoking and alcohol consumption with respect to serum gamma-glutamyltransferase. Hepatology 2009;49(3):802-8.

**LCCS:**

Lightfoot TJ, Barrett JH, Bishop T, Northwood EL, Smith G, Wilkie MJ, Steele RJ, Carey FA, Key TJ, Wolf R, and Forman D. Methylene tetrahydrofolate reductase genotype modifies the chemopreventive effect of folate in colorectal adenoma, but not colorectal cancer. *Cancer Epidemiol Biomarkers Prev* 2008;17:2421-2430.

Turner F, Smith G, Sachse C, Lightfoot T, Garner RC, Wolf CR, Forman D, Bishop DT, and Barrett JH. Vegetable, fruit and meat consumption and potential risk modifying genes in relation to colorectal cancer. *Int J Cancer* 2014;112:259-264.

**MCCS:**

Giles GG, English DR. The Melbourne Collaborative Cohort Study. IARC Sci Publ 2002;156:69-70.

**MEC:**

Kolonel LN, Henderson BE, Hankin JH, et al. A multiethnic cohort in Hawaii and Los Angeles: baseline characteristics. Am J Epidemiol 2000;151:346–357.

**MECC:**

Poynter JN, Gruber SB, Higgins PD*, et al.* Statins and the risk of colorectal cancer. N Engl J Med 2005;352(21):2184-92.

**NCCCSI/II:**

Keku TO, Vidal A, Oliver S, Hoyo C, Hall IJ, Omofoye O, McDoom M, Worley K, Galanko J, Sandler RS, and Millikan R. Genetic variants in IGF-I, IGF-II, IGFBP-3, and adiponectin genes and colon cancer risk in African Americans and Whites. *Cancer Causes Control* 2012;23:1127-1138.

Vinikoor LC, Long MD, Keku TO, Martin CF, Galanko JA, and Sandler RS. The association between diabetes, insulin use, and colorectal cancer among Whites and African Americans. *Cancer Epidemiol Biomarkers Prev* 2009;18:1239-1242.

**NHS:**

Belanger CF, Hennekens CH, Rosner B, and Speizer FE. The nurses' health study. *Am J Nurs.* 1978;78:1039-1040.

**PLCO:**

Huang J, Mondul AM, Weinstein SJ, Koutros S, Derkach A, Karoly E, Sampson JN, Moore SC, Berndt SI, and Albanes D. Serum metabolomic profiling of prostate cancer risk in the prostate, lung, colorectal, and ovarian cancer screening trial. *Br J Cancer* 2016;115:1087-1095.

**USC_HRT_CRC:**

Wu AH, Siegmund KD, Long TI*, et al.* Hormone therapy, DNA methylation and colon cancer. Carcinogenesis 2010;31(6):1060-7.

**VITAL:**

White E, Patterson RE, Kristal AR, et al. VITamins And Lifestyle cohort study: study design and characteristics of supplement users. Am J Epidemiol 2004; 159:83–93.

**WHI:**

Hays J, Hunt JR, Hubbell FA, et al. The Women’s Health Initiative recruitment methods and results. Ann Epidemiol 2003; 13: S18–S77.

The Women’s Health Initiative Study Group. Design of the Women’s Health Initiative clinical trial and observational study. Control Clin Trials 1998; 19:61–109.

Bergstralh EJ, Kosanke JL. Computerized matching of cases to controls. 56th ed. Rochester MN: Department of Health Sciences Research, Mayo Clinic, 1995.
